# Construction of a circRNA-miRNA-mRNA Regulatory Network for Coronary Artery Disease by Bioinformatics Analysis

**DOI:** 10.1155/2022/4017082

**Published:** 2022-02-16

**Authors:** Zebo Zhang, Haiyan Qian, Li Wang, Zhenbo Tao, Keai Cheng, Kaiyue Wang, Yanqing Xie, Lina Zhang

**Affiliations:** ^1^Department of Preventative Medicine, Zhejiang Key Laboratory of Pathophysiology, Medical School of Ningbo University, Ningbo, Zhejiang Province 315211, China; ^2^Insitute of Geriatrics, The Affiliated Hospital of Medical School, Ningbo University, Ningbo, Zhejiang Province 315100, China

## Abstract

**Background:**

Circular RNAs (circRNAs) were known to be related to the pathogenesis of many diseases through competing endogenous RNA (ceRNA) regulatory mechanisms. However, the function of circRNA in coronary artery disease (CAD) remains unclear. In this study, we aim to construct a circRNA-related competing endogenous RNA (ceRNA) network in CAD.

**Methods:**

The gene expression profiles of CAD were obtained from Gene Expression Omnibus datasets. Bioinformatics analysis was performed to construct a ceRNA regulatory network, from which the hub genes involved were identified through protein-protein interaction (PPI) networks leading to the identification of the circRNA-miRNA-hub gene subnetwork. In addition, function enrichment analysis was performed to detect the potential biological mechanism in which circRNA might be involved.

**Results:**

A total of 115 DEcircRNAs (differentially expressed circRNAs), 17 DEmiRNAs (differentially expressed microRNAs), and 790 DEmRNAs (differentially expressed mRNAs) were identified between CAD and control groups from microarray datasets. Functional enrichment analysis showed that DEmRNAs were significantly involved in inflammation-related pathways and ubiquitin-protein ligase binding. After identifying 20 DEcircRNA-DEmiRNA pairs and 561 DEmiRNA-DEmRNA pairs, we obtained a circRNA-miRNA-mRNA regulatory network. PPI network analysis showed that eight hub genes were closely related to CAD, leading to the identification of a circRNA-miRNA-hub gene subnetwork consisting of nine circRNAs (hsa_circ_0020275, hsa_circ_0020387, hsa_circ_0020417, hsa_circ_0045512, hsa_circ_0047336, hsa_circ_0069094, hsa_circ_0071326, hsa_circ_0071330, and hsa_circ_0085340), four miRNAs (hsa-miR-136-5p, hsa-miR-376c-3p, hsa-miR-411-5p, and hsa-miR-654-5p), and eight mRNAs (MKRN1, UBE2H, UBE2W, UBE2D1, UBE2F, BE2J1, ZNRF1, and SIAH2). In addition, we discovered these hub genes were enriched in the ubiquitin-mediated proteolysis pathway, suggesting circRNAs may be involved in the pathogenesis of CAD through this pathway.

**Conclusions:**

This study may deepen our understanding of the potential role of circRNA-miRNA-mRNA regulation network in CAD and suggest novel diagnostic biomarkers and therapeutic targets for CAD.

## 1. Introduction

Coronary artery disease (CAD), a cardiac disease caused by abnormal structure or function of the coronary artery, remains one of the leading causes of death and poses a heavy socioeconomic burden worldwide [1]. Patients who were diagnosed with coronary artery disease often have pathological changes in coronary arteries or myocardium before taking medication or surgical treatment due to the insidious onset of CAD. At present, drug treatment and interventional therapy have greatly improved the clinical prognosis of patients with CAD; however, about one-fifth of patients with acute coronary syndromes will experience recurrence within a relatively short period of time (≤5 years) [2]. Therefore, it is of great importance to identify molecular biomarkers for the early diagnosis and prognostic evaluation of CAD as well as to discover new therapeutic targets for CAD.

Protein-coding genes make up less than 2% of the human genome, while the majority of transcripts are noncoding RNAS (ncRNAs) [3]. Circular RNA (circRNA), a kind of ncRNA, consists of continuous covalently closed loops without the 3′-poly-A tail and the 5′-cap structure, which enables it to resist degradation by ribonuclease, and thus has relative conservation and stability [4]. circRNAs are involved in the regulation of many physiological and pathological processes, affecting the occurrence and development of certain diseases [5]. Previous studies have shown that circRNAs, as competitive endogenous RNAs (ceRNAs), can act as microRNA (miRNA) sponges with miRNA response elements (MRE), to regulate miRNA expression, thereby regulating the target genes of miRNAs and participating in the pathogenesis of CAD [6]. For instance, by acting as a miR-652-3p sponge, circ_RUSC2 can enhance the migration and proliferation of coronary vascular smooth muscle cells (VSMCs) and prevent cells from apoptosis by increasing the expression of spleen tyrosine kinase (SYK), which is a downstream target of miR-661 [7]. Although some circRNAs have been found to be involved in the pathogenesis of CAD, the underlying mechanism of circRNAs in CAD still needs further research.

Here, in order to study the potential role of circRNA in the CAD, especially the circRNA-miRNA-mRNA regulatory network, we constructed a regulatory network consisting of 16 circRNAs, 6 miRNAs, and 267 mRNAs through bioinformatics analysis of multiple sets of the Gene Expression Omnibus (GEO) database. We used protein-protein interaction (PPI) networks to screen for hub genes and then constructed a circRNA-miRNA-hub gene subnetwork to further clarify the role of circRNA in CAD. Functional enrichment analysis was performed on these hub genes to understand the potential functional mechanisms of circRNAs. This study will provide in-depth insights into the potential pathogenesis of CAD and also suggest novel biomarkers and targets for CAD.

## 2. Methods

### 2.1. Data Collection

The microarray datasets used in the current study were acquired from the NCBI GEO database (http://www.ncbi.nlm.nih.gov/gds/). The circRNA expression data of CAD was obtained from GSE115733 [8], which included 24 CAD patients and 7 controls. The miRNA and mRNA expression data of CAD were derived from GSE59421 [9] (including 33 CAD patients and 37 controls) and GSE97320 [10] (including 3 CAD patients and 3 controls), respectively. The elemental details of these microarray datasets are presented in Table 1.

### 2.2. Identification of Differentially Expressed circRNAs, miRNAs, and mRNAs

After downloading raw microarray data, normalization and logarithmic methods were performed to preprocess the data. The ID of the corresponding probe name was converted into an international standard name. When multiple probes correspond to the same gene symbol, the final probe expression value is calculated by the maximum value of probes. The probes that did not match a gene symbol were deleted. The Bioconductor Limma package was used to identify the DEcircRNAs (differentially expressed circRNAs), DEmiRNAs (differentially expressed miRNAs), and DEmRNAs (differentially expressed mRNAs) between CAD samples and the control samples. The criteria for selection of DEcircRNAs and DEmRNAs were *P* value <0.05 and |log2(fold change [FC])| >1 (|FC| > 2). DEmiRNAs were identified with the criteria of *P* value <0.05 and |log2FC| >0.263 (|FC| > 1.2).

### 2.3. Functional and Pathway Enrichment Analysis

To explore the potential biological mechanisms in the occurrence and development of CAD, Gene Ontology (GO) and Kyoto Encyclopedia of Genes and Genomes (KEGG) pathway enrichment analyses were conducted by using the Database for Annotation, Visualization, and Integrated Discovery (DAVID, https://david.ncifcrf.gov/). GO terms include biological process (BP), cellular component (CC), and molecular function (MF). A P value less than 0.05 was set as the cut-off criterion.

### 2.4. Prediction of circRNA-miRNA and miRNA-mRNA Interactions

The circBank database (http://www.circbank.cn/) [11], a comprehensive database of human circRNA that has the feature of predicted binding miRNA, was used to obtain the possible interactions between the DEcircRNAs and DEmiRNAs. The miRDIP online tool (http://ophid.utoronto.ca/mirDIP/) [12], as an integrated database of human miRNA-target predictions across 30 independent resources with an integrative score, was used to identify the possible interactions between the DEmiRNAs and DEmRNAs via bidirectional mode search under a high confidence filter (score class 5% = high).

### 2.5. Construction of the circRNA-miRNA-mRNA Network

By integrating the circRNA-miRNA pairs and the miRNA-mRNA pairs, we constructed a circRNA-miRNA-mRNA regulation network. The nodes that cannot complete a circRNA-miRNA-mRNA axis were removed. As circRNAs could competitively bind miRNA to regulate mRNA expression [13], only the pairs of circRNA-miRNA and miRNA-mRNA with the reverse expression patterns were saved. Cytoscape (version 3.8.2) was used to visualize the circRNA-miRNA-mRNA network.

### 2.6. PPI Network Analysis

The Retrieval of Interacting Genes/Proteins (STRING) database (https://string-db.org/) [14], an interactive gene/protein database retrieval tool, was used to predict the associations between target genes in regulatory network analysis. Text mining, experiments, databases, coexpression, neighborhood, gene fusion, and co-occurrence were selected under the options of active interaction sources. The minimum required interaction score was set to medium confidence (score 0.4) in this study. The PPI network analysis results were visualized by the Cytoscape (version 3.8.2) software. The Molecular Complex Detection (MCODE) program, a plug-in of Cytoscape, was used to identify the hub gene clusters in the PPI network. The clusters that contained ≥5 nodes with MCODE scores ≥5 were set as cutoff criteria with the default parameters (degree cutoff ≥ 2, node score cutoff ≥ 0.2, K-core ≥ 2, and max depth = 100). In this study, the genes in the cluster were considered hub genes.

### 2.7. Reconstruction of the circRNA-miRNA-Hub Gene Subnetwork

To identify the association between DEcircRNAs, DEmiRNAs, and hub genes, we mapped the hub genes into the previously identified circRNA-miRNA-mRNA network and extracted the relevant DEcircRNAs and DEmiRNAs, based on which the circRNA-miRNA-hub gene subnetwork was identified. Cytoscape was used to visualize the circRNA-miRNA-hub gene subnetwork.

## 3. Results

### 3.1. Identification of DEmRNAs, DEmiRNAs, and DEcircRNAs

**Figure 1:**
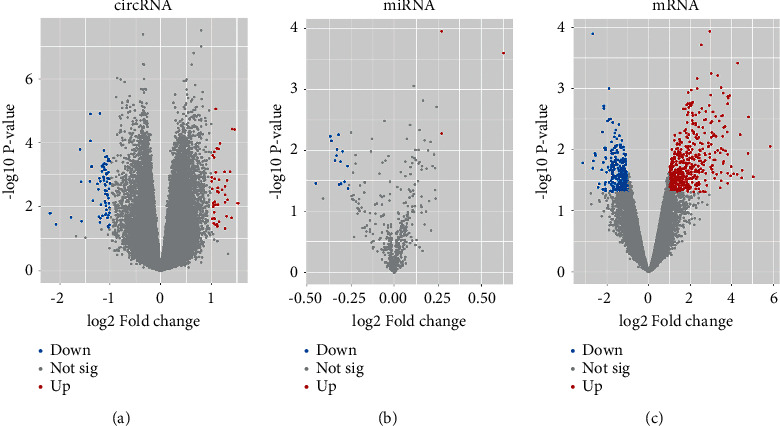
Volcano plots for each microarray. (a) Volcano plots of DEcircRNAs based on GSE115733. (b) Volcano plots of DEmiRNAs based on GSE59421. (c) Volcano plots of DEmRNAs based on GSE97320. DEcircRNAs, differentially expressed circular RNAs; DEmiRNAs, differentially expressed microRNAs; DEmRNAs, differentially expressed mRNAs.

A total of 115 DEcircRNAs (including 45 upregulated and 70 downregulated) and 790 DEmRNAs (including 496 upregulated and 294 downregulated) were identified from GSE115733 and GSE97320, respectively (Figures 1(a) and 1(b)). In addition, a total of 17 DEmiRNAs (3 upregulated and 14 downregulated) were identified from GSE59421 (Figure 1(c)). The complete DEcircRNAs, DEmiRNAs, and DEmRNAs identified in this study are listed in Tables S1–S3.

### 3.2. Functional Enrichment Analysis

**Figure 2:**
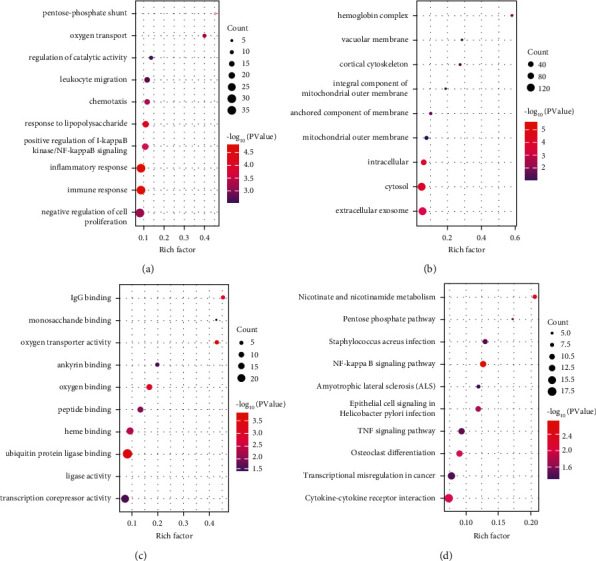
Thetop ten GO and KEGG enrichment terms for the differentially expressed mRNAs. (a) Biological process (BP), (b) cellular component (CC), (c) molecular function (MF), and (d) KEGG pathway. *Note*. The size of the circle represents gene counts. The color shows the *P* value, and the horizontal bar represents the corresponding *P* values. GO, Gene Ontology; KEGG, Kyoto Encyclopedia of Genes and Genomes.

GO and KEGG pathway enrichment analyses were performed on the 790 DEmRNAs identified in the previous section. The GO analysis demonstrated that the DEmRNAs were enriched in immune response (GO:0006955), inflammatory response (GO:0006954), and response to lipopolysaccharide (GO:0032496) for BP. When considering CC, hemoglobin complex (GO:0005833), cytosol (GO:0005829), and intracellular (GO:0005622) were the top three significantly enriched terms. With regard to MF, oxygen transporter activity (GO:0005344), ubiquitin-protein ligase binding (GO:0031625), and IgG binding (GO:0019864) were the most enriched terms. The top ten and total enriched terms are shown in Figures 2(a)–2(c) and Table S4, respectively. The KEGG pathway enrichment analysis indicated that the nicotinate and nicotinamide metabolism pathway (hsa: 00760) had the highest rich factor score. The top ten and total enriched KEGG pathways are shown in Figure 2(d) and Table S5, respectively.

### 3.3. Construction of the circRNA-miRNA-mRNA Network

**Figure 3:**
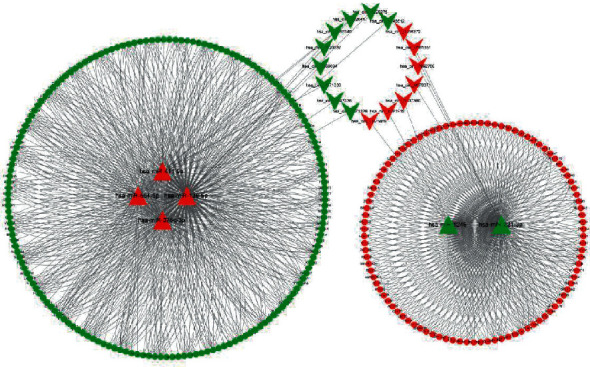
The circRNA-miRNA-mRNA regulatory network in coronary artery disease. The shape of V represents circRNAs, triangle represents miRNA, and ellipse represents mRNA. The red and green indicate upregulated expression and downregulated expression, respectively. The lines represent the connection between each other. circRNAs, circular RNAs; miRNAs, micro-RNAs.

A total of 20 DEcircRNA-DEmiRNA pairs and 561 DEmiRNA-DEmRNA pairs were identified through bioinformatics prediction. As shown in Figure 3, the circRNA-miRNA-mRNA network is composed of 16 circRNA nodes, 6 miRNA nodes, 267 mRNA nodes, and 1122 edges and was built based on the circRNA-miRNA and miRNA-mRNA pairs.

### 3.4. PPI Network Analysis

**Figure 4:**
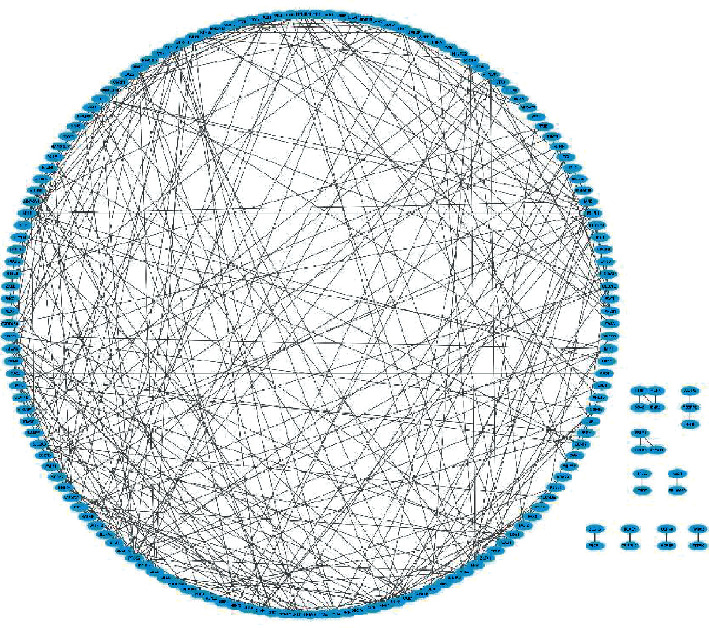
The PPI network based on the 267 target DEmRNAs in coronary artery disease. This network contained 173 nodes and 277 edges. PPI, protein-protein interaction; DEmRNAs, differentially expressed mRNA.

**Figure 5:**
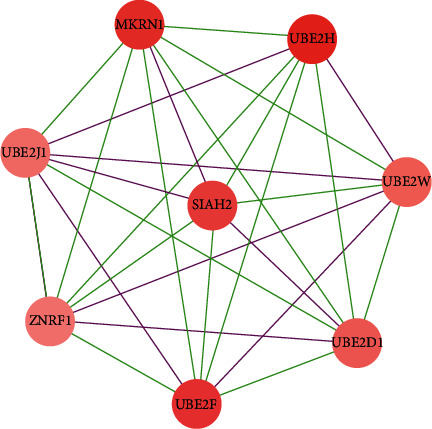
Hub genes extracted by MCODE plugin from the PPI network. MCODE, Molecular Complex Detection; PPI, protein-protein interaction.

The STRING database was used to construct a PPI network based on the 267 DEmRNAs with the unconnected nodes removed (Figure 4). The PPI network contained 173 nodes and 277 edges. By utilizing the algorithm of MCODE, only one cluster was selected with the criteria of MCODE scores of >5 and >5 nodes. Eight genes (MKRN1, UBE2H, UBE2W, UBE2D1, UBE2F, BE2J1, ZNRF1, and SIAH2) were identified as hub genes in this cluster, which contained eight nodes and 28 edges (Figure 5).

### 3.5. Construction and Analysis of the circRNA-miRNA-Hub Gene Subnetwork

**Figure 6:**
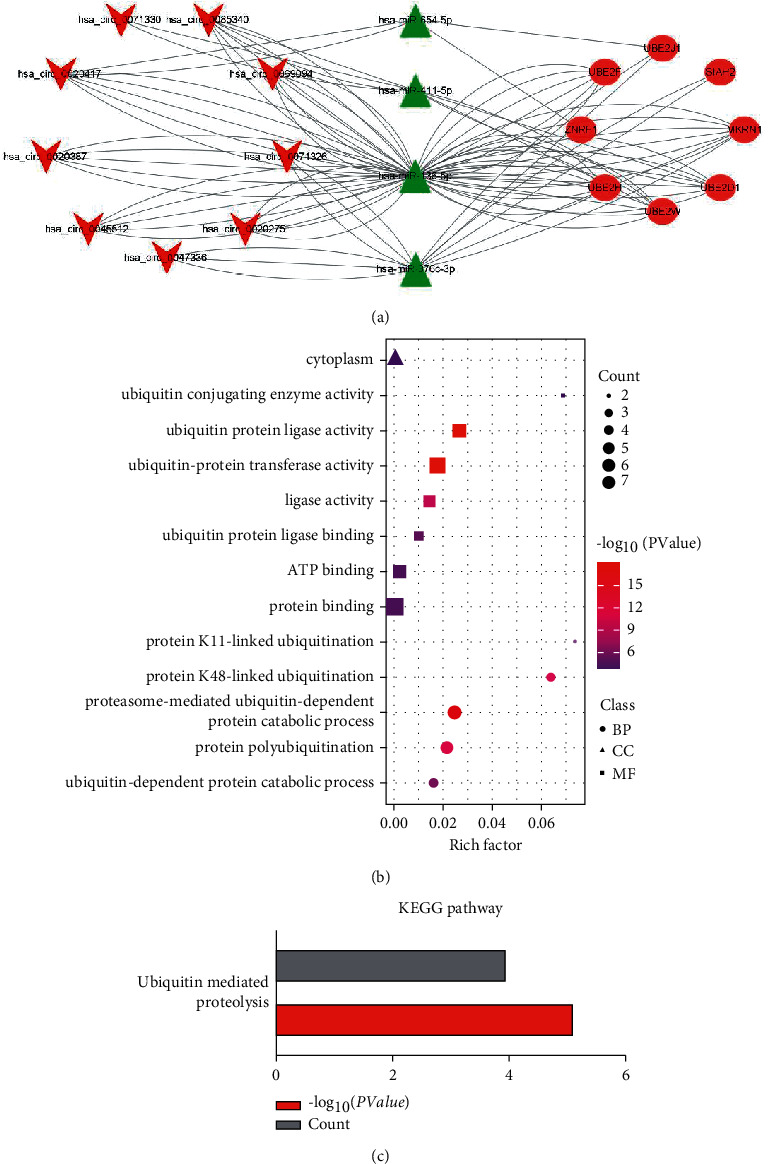
The circRNA-miRNA-hub gene regulatory subnetwork and functional enrichment analysis of the hub genes. (a) circRNA-miRNA-hub gene regulatory subnetwork. The shape of V represents circRNAs, triangle represents miRNA, and ellipse represents mRNA. The red and green indicate upregulated expression and downregulated expression, respectively. (b) GO enrichment analysis. *Note*. Circle represents BP, square represents MF, and triangle represents CC. The size of shapes represents gene counts. The color shows the *P* value, and the horizontal bar represents the corresponding *P* values. (c) KEGG pathway analysis. The abbreviations are the same as in Figure 2.

By mapping the eight hub genes into the previously identified circRNA-miRNA-mRNA network and extracting relevant circRNAs and miRNAs, a circRNA-miRNA-hub genes subnetwork was constructed. This subnetwork is consisted of nine circRNAs (hsa_circ_0020275, hsa_circ_0020387, hsa_circ_0020417, hsa_circ_0045512, hsa_circ_0047336, hsa_circ_0069094, hsa_circ_0071326, hsa_circ_0071330, and hsa_circ_0085340), four miRNAs (hsa-miR-136-5p, hsa-miR-376c-3p, hsa-miR-411-5p, and hsa-miR-654-5p), and eight mRNAs (MKRN1, UBE2H, UBE2W, UBE2D1, UBE2F, BE2J1, ZNRF1, and SIAH2) (Figure 6(a) and Table S5). The basic information of circRNAs involved in the subnetwork is shown in Table 2. GO and KEGG pathway enrichment analyses were also performed on the eight hub genes involved in the subnetwork to determine their potential functions. Enrichment analysis revealed that the hub genes were implicated in 13 GO terms (such as ubiquitin-conjugating enzyme activity, ubiquitin-protein ligase activity, and ubiquitin-protein transferase activity) and one pathway (ubiquitin-mediated proteolysis) (Figures 6(b) and 6(c) and Table S6).

## 4. Discussion

CAD is a complex and multifactorial disorder with high mortality and morbidity worldwide [15]. The underlying mechanism of its occurrence and development remains largely unknown. Previously, many scholars have conducted in-depth studies on the pathogenesis of CAD with most of them focused on the protein-coding genes. In recent years, ncRNAs have become a research hotspot and more and more ncRNAs, especially circRNAs, have been found to have specific biological functions in the occurrence and development of diseases [6]. Compared to linear RNAs and other ncRNAs, circRNAs are more stable due to their circular structure, which makes circRNAs potentially important transcriptional regulators and therefore promising diagnostic markers [4]. The main biological function of circRNAs is that they can act as miRNA sponges, thereby alleviating the inhibition of miRNA on downstream target genes and upregulating the expression of target genes [13]. Previously, some researchers had performed circRNA microarray analysis in the tissue or peripheral blood of patients with CAD and found that some circRNAs played important roles in the pathogenesis of CAD [8, 16, 17]. However, as our understanding of circRNAs is still the tip of the iceberg, the exact role of circRNAs in CAD remains largely unclear. Hence, we attempt to identify a few circRNAs which may act as miRNA sponges to regulate the expression of downstream genes in CAD pathogenesis.

In this study, a total of 115 DEcircRNAs, 17 DEmiRNAs, and 790 DEmRNAs between CAD and control groups were identified from three different microarray datasets in the GEO database, respectively. Moreover, we performed functional analysis of these DEmRNAs, and the results showed that these DEmRNAs were enriched in inflammation-related pathways such as the nuclear factor-kappa B (NF-*κ*B) signaling pathway and the tumor necrosis factor (TNF) signaling pathway. It is well known that inflammation response plays an important role in the initiation and progression of CAD, as well as in coronary plaque instability [18]. Blunting the classical inflammatory cascade could reduce the risk of adverse events related to CAD [19]. As the master regulator of systematic inflammation, aberrant NF-*κ*B activation has been considered as a key step in the progression of atherosclerosis [20]. TNF, a proinflammatory cytokine produced by macrophages, was also a central regulator of the inflammatory response and may be involved in the pathogenesis of atherosclerosis through its effects on endothelial cell function, lipid metabolism, and enhanced vascular inflammatory response [21]. Zhou et al. disclosed that some inflammatory factors were upregulated within vascular endothelial cells under the stimulation of pathogens, which was mediated by TNF-*α* and NF-*κ*B signaling pathways [22]. Previous studies have shown that TNF-*α* and NF-*κ*B were significantly downregulated in CAD patients in Pakistan [23]. All the evidence supports our discovery that DEmRNAs may be involved in the progression of CAD through regulating inflammation response.

According to the ceRNA theory, circRNAs could competitively bind miRNA to regulate mRNA expression [13]. We predicted the interactions between the RNAs and then constructed a circRNA-miRNA-mRNA regulatory network via bioinformatics approaches. We utilized PPI networks and then screened out eight hub genes, including MKRN1, UBE2H, UBE2W, UBE2D1, UBE2F, BE2J1, ZNRF1, and SIAH2. MKRN1, an E3 ubiquitin ligase, plays a significant role in regulating disorders through the ubiquitination of substrate proteins. Bai et al. found that MKRN1 could promote p21 protein ubiquitination and the proteasome pathway degradation to negatively regulate p21 expression, thereby preventing intermittent hypoxia-induced myocardial apoptosis [24]. Kotla et al. found that MKRN1 expression was inhibited by TERF2IP S205 phosphorylation and it promoted endothelial cell activation and senescence, contributing to the development of atherogenesis [25]. UBE2H, UBE2W, UBE2D1, and UBE2F were part of the ubiquitin-conjugating enzyme E2 (UBE2) family, which was vital in the ubiquitin-proteasome system (UPS) [26]. UPS is an adenosine triphosphate (ATP)-dependent proteolysis pathway that degrades proteins in cells and membranes [27]. As UPS is essential for maintaining the balance between protein synthesis and degradation in cells, its dysfunction may be the cause of many pathological events, including CAD [28]. Chen et al. have found that the expression of ubiquitin in lymphocytes and monocytes isolated from patients with CAD was higher than that in healthy controls, and the expression of ubiquitin increased with the increase of severity of CAD [29]. In patients with acute coronary syndrome, ubiquitin immunoreactivity was high in unstable coronary plaques, suggesting UPS plays a pivotal role in the instability and rupture of coronary atherosclerotic plaques [30]. ZNRF1, a ring-type E3 ubiquitin ligase, could promote caveolin-1 ubiquitination and degradation to modulate inflammation through the UPS pathway [31]. In myocardial infarction, hypoxia activates SIAH2, which ubiquitinates AKAP121 and leads to its proteolysis. Interestingly, the downregulation of AKAP121 will decrease protein kinase A-dependent BAD and Drp1 phosphorylation, triggering their interaction with Bcl2 and Fis1, respectively, which enhances mitochondrial fission, mitochondrial ROS production, oxidative stress, cardiomyocyte death, and myocardial dysfunction [32, 33].

After mapping the eight hub genes into the preliminary circRNA-miRNA-mRNA network, a circRNA-miRNA-hub gene subnetwork was constructed. Four miRNAs (hsa-miR-136-5p, hsa-miR-376c-3p, hsa-miR-411-5p, and hsa-miR-654-5p) were identified in this subnetwork. The reports of these miRNAs have mainly focused on tumors. hsa-miR-136-5p was significantly downregulated in several types of cancers and functions as tumor suppressor. Zhu et al. revealed that hsa-miR-136-5p was associated with impaired tumorigenesis and metastasis in prostate cancer by targeting MAP2K4 [34]. hsa-miR-376c-3p is also a tumor suppressor and plays an important role in the inhibition of gastric tumor growth and tumor-related gene expression both in vitro and in vivo [35]. Studies have reported that hsa-miR-411-5p functions as a negative tumor regulator in ovarian cancer cells, displaying the potential of miR-411-5p as a biomarker for ovarian cancer [36]. Han et al. found that hsa-miR-654-5p was identified as the potentially critical biomarker for atherosclerosis [37]. Our data indicated that these four miRNAs may act as regulatory factors and affect downstream target mRNA expression, thus participating in the occurrence and development of CAD.

Recent studies have shown that the circRNA-miRNA-mRNA regulatory network could identify the disease-related circRNAs and potential axis [38]. Miao et al. established a triple regulatory network of circRNA-miRNA-mRNA and found circ-YOD1 could be a good marker to predict the onset of CAD [39]. Mao et al. constructed a serum exosome-associated ceRNA network to identify the promising diagnostic and therapeutic targets for CAD [40]. Ji et al. performed RNA sequence analysis of circRNAs in peripheral blood mononuclear cells of patients with CAD and constructed a differentially expressed gene-circRNA-miRNA-mRNA network to identify dysregulated circRNAs involved in the pathogenesis of CAD [41]. In this study, we identified nine circRNAs (hsa_circ_0020275, hsa_circ_0020387, hsa_circ_0020417, hsa_circ_0045512, hsa_circ_0047336, hsa_circ_0069094, hsa_circ_0071326, hsa_circ_0071330, and hsa_circ_0085340) involved in the circRNA-miRNA-hub gene regulatory subnetwork. At present, none of the other nine circRNAs, except for hsa_circ_0069094, have been reported to be involved in the development of any diseases. hsa_circ_0069094 was found to upregulate HK2 expression by binding to miR-591, thus promoting cellular malignancies and glycolysis in breast cancer [42]. Another study revealed that hsa_circ_0069094 knockdown inhibited breast cancer cell glycolysis and cell carcinogenesis by regulating HMGA1 through sponging miR-661 [43]. Whether these circRNAs play a significant role in the pathogenesis of cardiovascular diseases, especially CAD, remains unclear. In order to clarify the potential role of circRNAs in CAD, we performed functional enrichment analysis for the hub genes, and the results indicated that these hub genes were enriched in the ubiquitin-mediated proteolysis pathway, which is consistent with the functional enrichment results of DEmRNAs. It suggests these circRNAs involved in the subnetwork may act as miRNA sponges to regulate the hub genes through ubiquitin-mediated proteolysis, thus participating in the pathogenesis of CAD.

In summary, our study constructed and analyzed a CAD-related circRNA-miRNA-mRNA network through integrated bioinformatics analysis, and we identified nine circRNAs that target the regulation of downstream genes through sponge-adsorbed miRNAs. However, there are still some limitations to this study. First, this is a retrospective study based on in silico data from the GEO database. All three datasets selected in this study were from different samples and different array platforms, which might have influenced the obtained conclusion. In addition, our results were mainly derived from bioinformatics predictions, and the proposed mechanism of circRNAs needed to be confirmed by laboratory studies. The advantage of our study is that it constructs a new circRNA-miRNA-mRNA network, which provides a new research direction for CAD. Based on this novel network, we further investigate the potential mechanism of circRNAs in CAD and identify potential biomarkers and therapeutic targets.

## 5. Conclusion

In this study, we identified nine circRNAs (hsa_circ_0020275, hsa_circ_0020387, hsa_circ_0020417, hsa_circ_0045512, hsa_circ_0047336, hsa_circ_0069094, hsa_circ_0071326, hsa_circ_0071330, and hsa_circ_0085340) may be involved in the pathogenesis of CAD by constructing a circRNA-miRNA-mRNA regulatory network. Optimistically, this study may deepen our understanding of the potential pathogenesis of CAD and also suggest novel biomarkers and targets for CAD.

## Figures and Tables

**Table 1:** Basic information of the microarray datasets from GEO.

Type	Data source	Platform	Samples (CAD/control)	Sample source	Year	Region	Author
circRNA	GSE115733	GPL22121	31 (24/7)	Peripheral blood	2018	China	Wang
miRNA	GSE59421	GPL10850	80 (33/37)	Peripheral blood	2014	The Netherlands	Maayke
mRNA	GSE97320	GPL570	6 (3/3)	Peripheral blood	2017	China	Meng

GEO, Gene Expression Omnibus; CAD, coronary artery disease; circRNA, circular RNA; miRNA, microRNA.

**Table 2:** Basic information of circRNAs involved in the subnetwork.

circRNA	Position	Gene symbol	Regulation
hsa_circ_0045512	Chr17:66980196–67020494	ABCA9	Up
hsa_circ_0085340	Chr8:113293402–113326281	CSMD3	Up
hsa_circ_0071326	Chr4:159045731–159048766	FAM198B	Up
hsa_circ_0020417	Chr10:128768965–129245809	DOCK1	Up
hsa_circ_0020387	Chr10:128768965–128823047	DOCK1	Up
hsa_circ_0020275	Chr10:124273706–124274424	HTRA1	Up
hsa_circ_0069094	Chr4:6698619–6698897	S100P	Up
hsa_circ_0071330	Chr4:159161450–159165501	TMEM144	Up
hsa_circ_0047336	Chr18:28666538–28673606	DSC2	Up

circRNA, circular RNA.

## Data Availability

The authors confirm that all data underlying the findings are fully available without restriction. All relevant data are accessible from the GEO database (http://www.ncbi.nlm.nih.gov/gds/). Processed data are available from the corresponding author, Lina Zhang (e-mail: zhanglina@nbu.edu.cn).

## Supplementary Materials

Supplementary Materials Table S1: DEcircRNAs between the CAD and control samples based on GSE115733. Table S2: DEmiRNAs between the CAD and control samples based on GSE59421. Table S3: DEmRNAs between the CAD and control samples based on GSE97320. Table S4: GO enrichment analysis of DEmRNAs. Table S5: KEGG pathway enrichment analysis of DEmRNAs. Table S6: complete list of circRNA-miRNA-hub gene subnetwork pairs. Table S7: functional enrichment analysis of hub genes. DEcircRNAs, differentially expressed circular RNAs; DEmiRNAs, differentially expressed microRNAs; DEmRNAs, differentially expressed mRNAs; CAD, coronary artery disease.
