# Supplementary material for: Construction of a circRNA-miRNA-mRNA Regulatory Network for Coronary Artery Disease by Bioinformatics Analysis
Source: Cardiol Res Pract. 2022 Feb 16;2022:4017082. doi: 10.1155/2022/4017082 (PMC8866025; doi:10.1155/2022/4017082)
Supplement: Supplementary Materials — Table S1: DEcircRNAs between the CAD and control samples based on GSE115733. Table S2: DEmiRNAs between the CAD and control samples based on GSE59421. Table S3: DEmRNAs between the CAD and control samples based on GSE97320. Table S4: GO enrichment analysis of DEmRNAs. Table S5: KEGG pathway enrichment analysis of DEmRNAs. Table S6: complete list of circRNA-miRNA-hub gene subnetwork pairs. Table S7: functional enrichment analysis of hub genes. DEcircRNAs, differentially expressed circular RNAs; DEmiRNAs, differentially expressed microRNAs; DEmRNAs, differentially expressed mRNAs; CAD, coronary artery disease. [file 4017082.f1.docx]

**Supplementary Materials**

| **Table S1.** DEcircRNAs between the CAD and control samples based on GSE115733. | | |
| --- | --- | --- |
| circRNA | logFC | *P*-Value |
| hsa_circ_0069094 | 1.524781775 | 0.007712102 |
| hsa_circ_0031891 | 1.458118548 | 3.84E-05 |
| hsa_circ_0083900 | 1.413816333 | 3.77E-05 |
| hsa_circ_0069215 | 1.401212399 | 0.022051329 |
| hsa_circ_0005322 | 1.367782925 | 0.000811382 |
| hsa_circ_0036541 | 1.32193611 | 0.00606738 |
| hsa_circ_0040412 | 1.306423098 | 0.001463421 |
| hsa_circ_0070184 | 1.304425441 | 0.020249012 |
| hsa_circ_0082478 | 1.27487682 | 0.00423697 |
| hsa_circ_0057340 | 1.274262935 | 0.007041584 |
| hsa_circ_0070330 | 1.267317888 | 0.002275886 |
| hsa_circ_0052828 | 1.265895836 | 0.047212939 |
| hsa_circ_0027758 | 1.257199595 | 0.000808111 |
| hsa_circ_0052046 | 1.21176005 | 0.00299717 |
| hsa_circ_0071330 | 1.172924037 | 0.000102027 |
| hsa_circ_0007752 | 1.149771368 | 0.0014538 |
| hsa_circ_0088731 | 1.148099596 | 0.029363408 |
| hsa_circ_0020387 | 1.140750607 | 0.000152658 |
| hsa_circ_0012881 | 1.134896602 | 0.003631019 |
| hsa_circ_0070797 | 1.130441417 | 0.002538589 |
| hsa_circ_0067953 | 1.126633551 | 0.008628615 |
| hsa_circ_0086373 | 1.115808327 | 0.003311455 |
| hsa_circ_0088177 | 1.113422876 | 0.040658156 |
| hsa_circ_0026004 | 1.112794574 | 0.036081088 |
| hsa_circ_0079593 | 1.109409413 | 0.000149189 |
| hsa_circ_0085340 | 1.091809384 | 0.000831496 |
| hsa_circ_0021508 | 1.088170778 | 0.008591233 |
| hsa_circ_0071326 | 1.085369685 | 8.64E-06 |
| hsa_circ_0021930 | 1.085036763 | 0.036732257 |
| hsa_circ_0062216 | 1.066919595 | 0.006868425 |
| hsa_circ_0020417 | 1.063958116 | 0.00135548 |
| hsa_circ_0001442 | 1.063883601 | 0.023657839 |
| hsa_circ_0088732 | 1.05886906 | 0.023306062 |
| hsa_circ_0020275 | 1.05449211 | 0.014805634 |
| hsa_circ_0021509 | 1.05351634 | 0.000291323 |
| hsa_circ_0071423 | 1.053462143 | 0.000190463 |
| hsa_circ_0047909 | 1.051411873 | 0.033153563 |
| hsa_circ_0045512 | 1.045318398 | 0.008834652 |
| hsa_circ_0025576 | 1.039684104 | 0.007787962 |
| hsa_circ_0047336 | 1.034381748 | 0.010004769 |
| hsa_circ_0069972 | 1.031781001 | 0.000253127 |
| hsa_circ_0057292 | 1.013993377 | 0.023988851 |
| hsa_circ_0066984 | 1.013899095 | 0.002804818 |
| hsa_circ_0070805 | 1.009220232 | 0.001605199 |
| hsa_circ_0053381 | 1.00246878 | 0.001209958 |
| hsa_circ_0008732 | -1.003943466 | 0.001884719 |
| hsa_circ_0017793 | -1.011107079 | 0.015557315 |
| hsa_circ_0037360 | -1.012842319 | 0.037003784 |
| hsa_circ_0058799 | -1.013706624 | 0.00037572 |
| hsa_circ_0076807 | -1.018072486 | 0.010750254 |
| hsa_circ_0058808 | -1.020423896 | 0.001353653 |
| hsa_circ_0080038 | -1.020861639 | 0.002783991 |
| hsa_circ_0003272 | -1.024810139 | 0.007687143 |
| hsa_circ_0081944 | -1.028901201 | 0.018545357 |
| hsa_circ_0081915 | -1.032633624 | 0.00032558 |
| hsa_circ_0056272 | -1.034243396 | 0.000840626 |
| hsa_circ_0024600 | -1.034914549 | 0.044253992 |
| hsa_circ_0035049 | -1.035439814 | 0.005405637 |
| hsa_circ_0019069 | -1.040943014 | 0.001280263 |
| hsa_circ_0027585 | -1.046368088 | 0.040713507 |
| hsa_circ_0058800 | -1.046984207 | 0.000370521 |
| hsa_circ_0039194 | -1.053629739 | 0.022394194 |
| hsa_circ_0073452 | -1.05691424 | 0.004377788 |
| hsa_circ_0034550 | -1.058073513 | 0.001264988 |
| hsa_circ_0025489 | -1.058604854 | 0.004091449 |
| hsa_circ_0006859 | -1.060992179 | 0.00027205 |
| hsa_circ_0086419 | -1.061210804 | 0.001635094 |
| hsa_circ_0017546 | -1.069355967 | 0.000480534 |
| hsa_circ_0017550 | -1.073653182 | 0.000966308 |
| hsa_circ_0003787 | -1.074249221 | 0.003148187 |
| hsa_circ_0017545 | -1.074498192 | 0.00090404 |
| hsa_circ_0042125 | -1.076229316 | 0.012183439 |
| hsa_circ_0060878 | -1.079632817 | 0.009080215 |
| hsa_circ_0018445 | -1.088938826 | 0.016472874 |
| hsa_circ_0058810 | -1.091360135 | 0.000273572 |
| hsa_circ_0061351 | -1.092520573 | 0.007302861 |
| hsa_circ_0058795 | -1.093153754 | 0.003950246 |
| hsa_circ_0012574 | -1.093685018 | 0.002129427 |
| hsa_circ_0063643 | -1.095675022 | 0.017544848 |
| hsa_circ_0017549 | -1.099135325 | 0.000693122 |
| hsa_circ_0067768 | -1.104165073 | 0.001976239 |
| hsa_circ_0074350 | -1.108061531 | 0.003043947 |
| hsa_circ_0084233 | -1.10833734 | 0.006702626 |
| hsa_circ_0008732_T_R | -1.116497791 | 0.000679653 |
| hsa_circ_0017548 | -1.118540682 | 0.0004531 |
| hsa_circ_0003480 | -1.119976707 | 0.000173686 |
| hsa_circ_0008732_T_L | -1.12093595 | 0.001468169 |
| hsa_circ_0017547 | -1.123012609 | 0.000572544 |
| hsa_circ_0070970 | -1.124823161 | 0.001308875 |
| hsa_circ_0027732 | -1.135695626 | 0.001162772 |
| hsa_circ_0002329 | -1.139334087 | 0.000476149 |
| hsa_circ_0015862 | -1.155350225 | 0.019421765 |
| hsa_circ_0058811 | -1.164012632 | 0.000527602 |
| hsa_circ_0062706 | -1.167937206 | 0.000769002 |
| hsa_circ_0002977 | -1.189156481 | 0.008467882 |
| hsa_circ_0032151 | -1.193854801 | 0.001575703 |
| hsa_circ_0070671 | -1.195126814 | 0.02312576 |
| hsa_circ_0017544 | -1.200784636 | 1.22E-05 |
| hsa_circ_0013240 | -1.208204669 | 0.031343339 |
| hsa_circ_0083440 | -1.220347858 | 0.001268433 |
| hsa_circ_0034597 | -1.22446709 | 0.020746925 |
| hsa_circ_0029034 | -1.231115116 | 0.002899947 |
| hsa_circ_0068443 | -1.251161064 | 0.001938869 |
| hsa_circ_0070896 | -1.345381117 | 0.006469754 |
| hsa_circ_0004568 | -1.365942169 | 0.000562127 |
| hsa_circ_0040646 | -1.379780145 | 0.000554198 |
| hsa_circ_0008273 | -1.394611475 | 8.76E-05 |
| hsa_circ_0073010 | -1.395133045 | 1.24E-05 |
| hsa_circ_0087200 | -1.404104134 | 0.001611882 |
| hsa_circ_0069996 | -1.568025869 | 0.028414026 |
| hsa_circ_0067734 | -1.576499048 | 0.001665774 |
| hsa_circ_0017543 | -1.595580393 | 0.000159248 |
| hsa_circ_0069997 | -1.782131909 | 0.021775313 |
| hsa_circ_0053283 | -2.08655777 | 0.036317933 |
| hsa_circ_0079596 | -2.191181925 | 0.015855635 |
| Abbreviations: circRNA, circular RNA; DEcircRNAs, differentially expressed circRNAs; CAD, coronary artery disease; FC, fold change. | | |

| **Table S2.** DEmiRNAs between the CAD and control samples based on GSE59421. | | |
| --- | --- | --- |
| miRNAs | logFC | *P-*Value |
| hsa-miR-1246 | 0.626664564 | 0.000251726 |
| hsa-miR-221 | 0.272854399 | 0.000112533 |
| hsa-miR-1274a | 0.272260022 | 0.005360548 |
| hsa-miR-376a | -0.267081111 | 0.042115607 |
| hsa-miR-1234 | -0.269835647 | 0.018106648 |
| hsa-miR-1281 | -0.287265319 | 0.032854647 |
| hsa-miR-539 | -0.299373492 | 0.010557153 |
| hsa-miR-323-3p | -0.307159663 | 0.035304085 |
| hsa-miR-337-5p | -0.311002853 | 0.015184268 |
| hsa-miR-136 | -0.321222026 | 0.035810375 |
| hsa-miR-299-3p | -0.323060234 | 0.005536985 |
| hsa-miR-411 | -0.330794995 | 0.009773243 |
| hsa-miR-136* | -0.335126175 | 0.011906668 |
| hsa-miR-376c | -0.34366245 | 0.014616087 |
| hsa-miR-654-5p | -0.364299595 | 0.006964536 |
| hsa-miR-431 | -0.369365178 | 0.005875336 |
| hsa-miR-133b | -0.409581531 | 0.060871734 |
| hsa-miR-1 | -0.450297553 | 0.034196137 |
| Abbreviations: miRNA, microRNA; DEmiRNAs, differentially expressed miRNAs; CAD, coronary artery disease; FC, fold change. | | |

| **Table S3.** DEmRNAs between the CAD and control samples based on GSE97320. | | |
| --- | --- | --- |
| mRNAs | logFC | *P-*Value |
| HBM | 5.826547174 | 0.00883633 |
| EPB42 | 5.011148028 | 0.027607112 |
| TUBB2A | 4.797134865 | 0.002910262 |
| HBD | 4.775607724 | 0.011608491 |
| SELENBP1 | 4.51329006 | 0.021506443 |
| ALAS2 | 4.480526904 | 0.029012958 |
| PDZK1IP1 | 4.406408065 | 0.005679556 |
| S100P | 4.282255127 | 0.000378276 |
| AHSP | 4.265544086 | 0.025295445 |
| TSPAN5 | 4.088229861 | 0.024962058 |
| SLC25A39 | 3.958974686 | 0.019055487 |
| SLC6A8 | 3.948335806 | 0.026787945 |
| TNFRSF10C | 3.893217483 | 0.001289598 |
| PROK2 | 3.820450168 | 0.001436838 |
| SLC4A1 | 3.809720662 | 0.035340391 |
| SNCA | 3.80787415 | 0.011919738 |
| IL1R2 | 3.805209507 | 0.001353294 |
| HBG2 | 3.796071672 | 0.024169597 |
| CXCR2 | 3.787089324 | 0.001752202 |
| ADIPOR1 | 3.771167477 | 0.005169465 |
| JAZF1 | 3.706097005 | 0.010560471 |
| FCGR3B | 3.685639519 | 0.004151412 |
| PI3 | 3.60812541 | 0.004366447 |
| BNIP3L | 3.606389023 | 0.015530992 |
| HBQ1 | 3.587927582 | 0.017026676 |
| CMTM2 | 3.586874973 | 0.001179744 |
| TRIM58 | 3.569624012 | 0.00999098 |
| KRT23 | 3.562254755 | 0.005954587 |
| KCNJ15 | 3.549343007 | 0.002724722 |
| MANSC1 | 3.499293137 | 0.002799885 |
| KLF1 | 3.492882626 | 0.032464964 |
| MGAM | 3.490587199 | 0.000961117 |
| STRADB | 3.481507487 | 0.02353179 |
| DCAF12 | 3.480749066 | 0.009568395 |
| CA1 | 3.435990311 | 0.042077605 |
| BAG1 | 3.421575948 | 0.011190847 |
| CYP4F3 | 3.383149714 | 0.003370019 |
| HCAR3 | 3.312410661 | 0.000605758 |
| TESC | 3.301006624 | 0.010198664 |
| ASCC2 | 3.270232927 | 0.025074062 |
| CHI3L1 | 3.231263507 | 0.005958946 |
| BLVRB | 3.219593399 | 0.002834527 |
| NFE2 | 3.190794114 | 0.011086403 |
| TENT5C | 3.187548116 | 0.014807251 |
| GYPC | 3.162554712 | 0.029214687 |
| FBXO7 | 3.141390642 | 0.014693785 |
| SLC25A37 | 3.129192789 | 0.00571075 |
| TNS1 | 3.123713856 | 0.005931915 |
| GUK1 | 3.094347623 | 0.023270281 |
| TNFAIP6 | 3.084674642 | 0.002405944 |
| NAMPT | 3.077736995 | 0.018089606 |
| LGALS3 | 3.051883106 | 0.02099861 |
| HBA2 | 3.045929379 | 0.005277123 |
| YBX3 | 3.033784666 | 0.003424214 |
| GMPR | 3.026846597 | 0.006211054 |
| PCGF5 | 3.026666891 | 0.008194251 |
| BASP1 | 3.022726509 | 0.000560725 |
| GPR27 | 3.004570806 | 0.00128572 |
| FFAR2 | 2.997665641 | 0.003622945 |
| FRAT2 | 2.993091586 | 0.019153451 |
| FAM210B | 2.949019512 | 0.01333901 |
| PTGS2 | 2.948947276 | 0.000116072 |
| RNF10 | 2.941844699 | 0.011841907 |
| CDA | 2.920600294 | 0.001861546 |
| UBXN6 | 2.895055994 | 0.008715013 |
| XK | 2.870352893 | 0.008943869 |
| EMC3 | 2.864430818 | 0.007733442 |
| ABCC13 | 2.78770292 | 0.0384538 |
| MMP9 | 2.76242004 | 0.003176423 |
| UBE2H | 2.756291293 | 0.024288313 |
| AQP9 | 2.748013003 | 0.002366251 |
| NINJ2 | 2.730337 | 0.015161396 |
| MXI1 | 2.727602505 | 0.031834448 |
| F2RL1 | 2.718877382 | 0.012184166 |
| NRBF2 | 2.716019041 | 0.049236911 |
| CSF2RB | 2.712866027 | 0.005344837 |
| HEMGN | 2.709780052 | 0.011520431 |
| VNN2 | 2.683242451 | 0.003258606 |
| PPM1A | 2.659716401 | 0.017889884 |
| GLRX5 | 2.653978801 | 0.016000128 |
| KRT1 | 2.628386302 | 0.048152465 |
| ALPL | 2.617818279 | 0.0217853 |
| BSG | 2.611648897 | 0.009702209 |
| IGF2BP2 | 2.595984533 | 0.004314693 |
| MARCHF8 | 2.587599362 | 0.022951155 |
| SLC22A4 | 2.575974351 | 0.007999812 |
| ACSL1 | 2.557449024 | 0.002103888 |
| WDR45 | 2.547644617 | 0.016811081 |
| ADM | 2.520038924 | 0.000195984 |
| DMTN | 2.507848171 | 0.015311555 |
| DPM2 | 2.495411764 | 0.020379627 |
| QPCT | 2.49249606 | 0.007744829 |
| STX17-DT | 2.491666613 | 0.049212837 |
| PYGL | 2.472955446 | 0.010406727 |
| CHPT1 | 2.469086722 | 0.010103583 |
| CCNDBP1 | 2.466576571 | 0.049581069 |
| YPEL3 | 2.439251755 | 0.023220982 |
| FECH | 2.43819572 | 0.015308258 |
| GSPT1 | 2.436003962 | 0.023277798 |
| MKRN1 | 2.435488323 | 0.012138332 |
| RNF149 | 2.434999859 | 0.036063001 |
| FKBP1B | 2.427697685 | 0.013804977 |
| SLPI | 2.425362314 | 0.002462863 |
| CDC42BPA | 2.416385737 | 0.044127261 |
| C5AR1 | 2.412944803 | 0.00627643 |
| MXD1 | 2.412657768 | 0.00428944 |
| FIS1 | 2.400880489 | 0.03806141 |
| FPR2 | 2.400051032 | 0.034003974 |
| MARCHF2 | 2.398536282 | 0.011914471 |
| SRRD | 2.398185061 | 0.0215739 |
| FLACC1 | 2.373490259 | 0.018460373 |
| OPTN | 2.358252787 | 0.022468656 |
| GUCD1 | 2.348467687 | 0.013296181 |
| NFIL3 | 2.343014488 | 0.000676191 |
| SLC38A5 | 2.335574 | 0.027970113 |
| STEAP4 | 2.333103645 | 0.001000438 |
| LYL1 | 2.329618061 | 0.006610299 |
| SEC14L1 | 2.327503659 | 0.0276414 |
| CARM1 | 2.32534393 | 0.022968465 |
| MME | 2.322053667 | 0.038922748 |
| PPP1R3B | 2.321042593 | 0.005631717 |
| RBM38 | 2.314922073 | 0.035352047 |
| EGLN1 | 2.300237575 | 0.037414763 |
| LILRA5 | 2.298935676 | 0.012829351 |
| NPRL3 | 2.283415099 | 0.030013555 |
| G0S2 | 2.278544604 | 0.012685171 |
| PLEK2 | 2.269759479 | 0.044012341 |
| STMP1 | 2.269317517 | 0.003687392 |
| ADGRG3 | 2.264723169 | 0.012850577 |
| MMP25 | 2.255506241 | 0.006185791 |
| CXCL8 | 2.253351525 | 0.002682615 |
| NIBAN1 | 2.252521712 | 0.02139803 |
| REPS2 | 2.251650417 | 0.003948461 |
| MCOLN1 | 2.251505934 | 0.012144939 |
| BCL2A1 | 2.241485059 | 0.017825766 |
| UBE2F | 2.235569001 | 0.037597894 |
| KCNJ2 | 2.207053265 | 0.027018427 |
| IFI27 | 2.206575757 | 0.008092199 |
| STX3 | 2.204695884 | 0.02636643 |
| TGM2 | 2.20303579 | 0.022921553 |
| CXCR1 | 2.182128454 | 0.014450755 |
| OSBP2 | 2.170975469 | 0.014140242 |
| SERPINA1 | 2.161118638 | 0.018613178 |
| B3GNT8 | 2.157266116 | 0.005497874 |
| BCL6 | 2.155436651 | 0.049233261 |
| GCA | 2.155221538 | 0.005115182 |
| LRG1 | 2.151975902 | 0.007893825 |
| MPP1 | 2.137607705 | 0.003647113 |
| CR1L | 2.132070419 | 0.028821534 |
| MBOAT7 | 2.126594648 | 0.002738999 |
| FPR1 | 2.121863904 | 0.003810309 |
| HAGH | 2.114650791 | 0.028116766 |
| CXCL1 | 2.109890678 | 0.00167364 |
| SIAH2 | 2.104606201 | 0.009860117 |
| FAM174A | 2.103570259 | 0.034395065 |
| DGAT2 | 2.103296687 | 0.002509107 |
| BCL2L1 | 2.0981445 | 0.013205171 |
| BPGM | 2.092198793 | 0.019830541 |
| ELAPOR1 | 2.088822219 | 0.019701271 |
| CYSTM1 | 2.088341024 | 0.00490145 |
| NFE4 | 2.086185009 | 0.003341197 |
| THBD | 2.074314967 | 0.001728655 |
| PRDX6 | 2.070007714 | 0.02052596 |
| MAP2K3 | 2.063033314 | 0.017588269 |
| EIF2AK1 | 2.059486892 | 0.014066939 |
| CDKN2D | 2.059069768 | 0.029959311 |
| MSRB1 | 2.049572992 | 0.00104864 |
| CLN8-AS1 | 2.048510433 | 0.023567321 |
| CTBS | 2.043283616 | 0.023052315 |
| NCF4 | 2.036124621 | 0.001878361 |
| MEGF9 | 2.032392257 | 0.025421184 |
| AK1 | 2.025182367 | 0.041460152 |
| RILP | 2.017698054 | 0.011160927 |
| CDC34 | 2.006201224 | 0.019388055 |
| FBXO9 | 1.999176923 | 0.029521484 |
| IL1B | 1.994257869 | 0.008836244 |
| TPGS2 | 1.993659067 | 0.020047972 |
| TREM1 | 1.986757197 | 0.001872506 |
| CDC27 | 1.983351056 | 0.040992987 |
| MICAL2 | 1.981552536 | 0.013661998 |
| TMCC3 | 1.978835017 | 0.007379451 |
| CXCL16 | 1.975013426 | 0.001775376 |
| R3HDM4 | 1.957823166 | 0.016692117 |
| CYB5R4 | 1.940846123 | 0.040174493 |
| FCGR2C | 1.937654603 | 0.011447894 |
| H4C8 | 1.93714733 | 0.043837962 |
| MNDA | 1.933132047 | 0.029979207 |
| TOLLIP | 1.932227823 | 0.005682311 |
| GRINA | 1.924431753 | 0.002020033 |
| TLR1 | 1.919744886 | 0.023705279 |
| NATD1 | 1.918620304 | 0.010010976 |
| PSMF1 | 1.912974981 | 0.014840352 |
| MAP1LC3B | 1.911581873 | 0.024874698 |
| GATA1 | 1.907083498 | 0.03738959 |
| CREB5 | 1.904269735 | 0.038967788 |
| IL1RN | 1.90192533 | 0.002421248 |
| SIRPB1 | 1.894300149 | 0.008864735 |
| RNF19B | 1.890727031 | 0.001149146 |
| AP2M1 | 1.888844498 | 0.0218779 |
| CEP19 | 1.885677069 | 0.037005383 |
| MUC8 | 1.883679332 | 0.046658062 |
| HMBS | 1.880191487 | 0.01678448 |
| NAPA | 1.879585081 | 0.009294363 |
| PILRA | 1.869536954 | 0.047990894 |
| VASP | 1.869258165 | 0.045946302 |
| IMPA2 | 1.869176084 | 0.006351885 |
| VNN3 | 1.86437749 | 0.001768857 |
| HHEX | 1.86369366 | 0.041524863 |
| ADGRE2 | 1.861318812 | 0.048724979 |
| RBM47 | 1.860959172 | 0.030615447 |
| WLS | 1.859856499 | 0.014398304 |
| LY96 | 1.846743665 | 0.002711577 |
| RNF24 | 1.845235298 | 0.024285488 |
| HAL | 1.840749749 | 0.003557837 |
| JDP2 | 1.834187231 | 0.027306688 |
| SOD2 | 1.828196898 | 0.001817764 |
| SIGLEC5 | 1.824835166 | 0.012598105 |
| ATP6V0C | 1.823689036 | 0.006130922 |
| GLT1D1 | 1.823477092 | 0.010929845 |
| GSEC | 1.814407351 | 0.011544649 |
| TNFRSF1A | 1.812886806 | 0.034248936 |
| FAM53A | 1.811754969 | 0.017490244 |
| LYN | 1.810079435 | 0.021983392 |
| PLXNC1 | 1.809156191 | 0.018616326 |
| PITHD1 | 1.801019653 | 0.042481047 |
| FUNDC2 | 1.798304401 | 0.038064421 |
| SDCBP | 1.796003771 | 0.005317856 |
| RRAGD | 1.785403999 | 0.008723657 |
| E2F2 | 1.780220175 | 0.028056126 |
| PGD | 1.779741082 | 0.01959234 |
| DCAF10 | 1.779434823 | 0.030269821 |
| KDM7A-DT | 1.773776539 | 0.004722006 |
| S100A11 | 1.76931377 | 0.016587279 |
| MSI2 | 1.768455455 | 0.040075517 |
| SMIM5 | 1.758195371 | 0.00212754 |
| CAT | 1.755742967 | 0.03783598 |
| CRISPLD2 | 1.737200431 | 0.004928536 |
| USP10 | 1.732881641 | 0.019674016 |
| BMP2K | 1.723610182 | 0.019605719 |
| RHOG | 1.713914764 | 0.043437285 |
| ORM1 | 1.705773409 | 0.03338373 |
| MBOAT2 | 1.698660154 | 0.020277511 |
| LARP1B | 1.695226279 | 0.027195476 |
| CCNJL | 1.687902542 | 0.012063108 |
| GPR160 | 1.684884685 | 0.035964519 |
| DNAH2 | 1.680998076 | 0.016545503 |
| BCL3 | 1.670172216 | 0.015156602 |
| GFUS | 1.659583819 | 0.024103495 |
| TNNT1 | 1.65320048 | 0.002123128 |
| NCF2 | 1.650504688 | 0.025252557 |
| AKIRIN2 | 1.64278317 | 0.038212556 |
| POLR1D | 1.63847307 | 0.045352032 |
| CSF2RA | 1.637856851 | 0.023231511 |
| GNA12 | 1.633652469 | 0.016342405 |
| FCGR1B | 1.632900598 | 0.025472145 |
| CSF3R | 1.632065062 | 0.029757973 |
| FAM86B3P | 1.624527698 | 0.020594773 |
| BST1 | 1.618049329 | 0.02657119 |
| P2RY13 | 1.617494114 | 0.013406886 |
| LINC01094 | 1.610011588 | 0.044569594 |
| RPIA | 1.607963197 | 0.029416128 |
| TFDP1 | 1.601010551 | 0.027513305 |
| ADAMTS10 | 1.593281392 | 0.032915859 |
| SRXN1 | 1.591124608 | 0.035582405 |
| CCDC71L | 1.590329707 | 0.0076108 |
| OAZ2 | 1.585432282 | 0.013225725 |
| WBP2 | 1.582826057 | 0.012179969 |
| ELOB | 1.577521906 | 0.024228482 |
| MED25 | 1.567389822 | 0.043956894 |
| ITPRIP | 1.5650322 | 0.021423471 |
| IRAG1 | 1.564721917 | 0.009812706 |
| TRIB1 | 1.562628262 | 0.038525263 |
| TMEM63B | 1.558659403 | 0.046375257 |
| RAD23A | 1.557590856 | 0.033872055 |
| FRAT1 | 1.551630032 | 0.045314574 |
| SLC2A3 | 1.549882492 | 0.025067178 |
| PGLYRP1 | 1.548053287 | 0.028117311 |
| TSEN34 | 1.547542336 | 0.015609173 |
| UBE2D1 | 1.542750891 | 0.032773663 |
| POLL | 1.541385336 | 0.022989059 |
| DNAJB2 | 1.528639981 | 0.023488484 |
| LITAF | 1.526662463 | 0.04306807 |
| BACH1 | 1.526295367 | 0.042245241 |
| UBE2W | 1.524678587 | 0.032847791 |
| RALB | 1.519423449 | 0.019731748 |
| FCGRT | 1.51828828 | 0.03509035 |
| CTSB | 1.517900297 | 0.016402528 |
| TALDO1 | 1.516049827 | 0.011422082 |
| KIFC3 | 1.511051006 | 0.034745102 |
| PPP2R5B | 1.510233831 | 0.045229431 |
| HLX | 1.506771962 | 0.021638345 |
| PNRC1 | 1.504586234 | 0.031182931 |
| ZYX | 1.504433409 | 0.023091288 |
| NINJ1 | 1.50358796 | 0.007268735 |
| UBL7 | 1.497729781 | 0.015944504 |
| SLC11A1 | 1.497362516 | 0.011116132 |
| DYSF | 1.495721297 | 0.005373425 |
| CLEC4E | 1.490314024 | 0.024192691 |
| SERF2 | 1.488080372 | 0.023387463 |
| TNFRSF12A | 1.479244296 | 0.028420121 |
| TNIP1 | 1.478671017 | 0.010780053 |
| LOC643072 | 1.476705093 | 0.011250833 |
| CEBPB | 1.474155503 | 0.03116714 |
| FAH | 1.473957596 | 0.008104714 |
| PRRG4 | 1.473492411 | 0.006710574 |
| GBGT1 | 1.470669995 | 0.014047492 |
| TAL1 | 1.466589262 | 0.042690394 |
| TCF15 | 1.464530736 | 0.030137407 |
| ABCB9 | 1.462317284 | 0.049456655 |
| TIMP2 | 1.457595808 | 0.02099547 |
| NKX1-1 | 1.456792367 | 0.031831817 |
| CCR10 | 1.455892866 | 0.01261541 |
| LCN2 | 1.453813459 | 0.038573672 |
| SNX3 | 1.447443267 | 0.028282022 |
| ELOF1 | 1.438345812 | 0.02584437 |
| PLIN3 | 1.437430663 | 0.011281192 |
| IFITM2 | 1.436777606 | 0.047745309 |
| KAZN | 1.435583412 | 0.037578889 |
| F11R | 1.433773308 | 0.049927708 |
| GLUL | 1.426209388 | 0.010570665 |
| EGFL7 | 1.415177005 | 0.009437636 |
| ARL4A | 1.414659872 | 0.030501581 |
| ASMTL-AS1 | 1.41103132 | 0.046493662 |
| SPI1 | 1.410521231 | 0.012266005 |
| DLG4 | 1.405291089 | 0.027537936 |
| SLC7A5 | 1.401334844 | 0.020835081 |
| MSRB2 | 1.399682346 | 0.021674389 |
| TMEM154 | 1.398948162 | 0.023736182 |
| TMEM86B | 1.395261695 | 0.048436836 |
| ROGDI | 1.392052805 | 0.027661024 |
| FCER1G | 1.383875473 | 0.049144038 |
| C1QC | 1.382419988 | 0.023922596 |
| SULT1B1 | 1.378755298 | 0.009480946 |
| GSN | 1.376904976 | 0.036261879 |
| PHC2 | 1.376695887 | 0.007899992 |
| YIPF3 | 1.376092222 | 0.028169202 |
| PIGX | 1.372086982 | 0.023767514 |
| TUBB3 | 1.370238523 | 0.017935517 |
| PCTP | 1.369372439 | 0.011855501 |
| SLC15A4 | 1.367281158 | 0.044836822 |
| BID | 1.366654577 | 0.047009965 |
| ZNF653 | 1.365680377 | 0.031181562 |
| TSHZ3 | 1.359599208 | 0.008145728 |
| CDC42EP2 | 1.348933887 | 0.032202603 |
| TMEM140 | 1.347831854 | 0.028916531 |
| LILRA2 | 1.346445432 | 0.01550573 |
| CYRIA | 1.344893511 | 0.009754982 |
| HBB | 1.34368003 | 0.048436107 |
| PINK1 | 1.339258876 | 0.046219659 |
| IFITM3 | 1.32612605 | 0.044959364 |
| ISLR2 | 1.322535139 | 0.040641788 |
| GIT1 | 1.321796709 | 0.031349571 |
| IL13RA1 | 1.31977459 | 0.017509747 |
| FOXO4 | 1.319401574 | 0.041015703 |
| ADAD2 | 1.318543357 | 0.011734825 |
| TAMALIN | 1.312899456 | 0.033473824 |
| SFRP2 | 1.310383127 | 0.037129785 |
| COL9A3 | 1.30749668 | 0.036247217 |
| UBB | 1.291662183 | 0.039020265 |
| WDR25 | 1.289622639 | 0.022776672 |
| GPX1 | 1.286594395 | 0.021402744 |
| SLC45A1 | 1.286331296 | 0.027190904 |
| PAK1 | 1.27721854 | 0.029951229 |
| CDKN2C | 1.27647096 | 0.043640541 |
| SMOX | 1.274509923 | 0.005748589 |
| SECTM1 | 1.27173976 | 0.010856219 |
| G6PD | 1.269978971 | 0.023415962 |
| IL1R1 | 1.268966265 | 0.005759851 |
| FURIN | 1.267871787 | 0.011077087 |
| IGFBP2 | 1.264461546 | 0.028399961 |
| TM4SF5 | 1.262983341 | 0.034625663 |
| UBE2J1 | 1.262885456 | 0.027866887 |
| RNF187 | 1.258896529 | 0.025341387 |
| ODC1 | 1.258416673 | 0.04953787 |
| ABTB1 | 1.258074984 | 0.013543545 |
| DOK3 | 1.254960594 | 0.014257424 |
| RNASE2 | 1.250637683 | 0.006959991 |
| DPCD | 1.248381655 | 0.044396156 |
| PADI2 | 1.247799737 | 0.011845346 |
| RNF14 | 1.241485235 | 0.023052401 |
| EMID1 | 1.241424461 | 0.029840999 |
| RETREG2 | 1.240836415 | 0.03961845 |
| FAM214B | 1.236148272 | 0.013221567 |
| FLOT2 | 1.235719004 | 0.023724411 |
| TANGO2 | 1.233441763 | 0.023693183 |
| ANKRD2 | 1.232814864 | 0.043486472 |
| NAPRT | 1.232160212 | 0.035117407 |
| FAM240C | 1.228684817 | 0.033620342 |
| PRCP | 1.224949673 | 0.041886065 |
| TLCD3B | 1.220039274 | 0.03137741 |
| THTPA | 1.217819638 | 0.02506633 |
| MAF1 | 1.211403679 | 0.023575937 |
| CHST11 | 1.201155813 | 0.038451237 |
| LTBR | 1.198553415 | 0.01598486 |
| PLAUR | 1.193391676 | 0.014202724 |
| AIRE | 1.187492543 | 0.018524588 |
| LRRC25 | 1.17915376 | 0.016318801 |
| S100A12 | 1.173768993 | 0.040774363 |
| NBAT1 | 1.172352668 | 0.047012642 |
| KCNE3 | 1.17028762 | 0.036024863 |
| RASGRP4 | 1.168028926 | 0.037248765 |
| ZDHHC22 | 1.167516392 | 0.041934893 |
| GID8 | 1.164530358 | 0.02702696 |
| ZNRF1 | 1.161008638 | 0.036287829 |
| CEACAM3 | 1.160509641 | 0.026765808 |
| LOC102724880 | 1.1598637 | 0.035119064 |
| WDR13 | 1.159258719 | 0.04158229 |
| FBXL8 | 1.15913223 | 0.015764577 |
| PCDHB11 | 1.156438022 | 0.035406597 |
| RNF123 | 1.156154574 | 0.0406258 |
| USP2 | 1.155540827 | 0.042917313 |
| SLC1A2 | 1.152087825 | 0.037704858 |
| DOCK4 | 1.150810307 | 0.019140814 |
| CTSD | 1.149156263 | 0.041812441 |
| MIR3667HG | 1.147963699 | 0.030794316 |
| LIN7A | 1.147732875 | 0.015985065 |
| LOC105372485 | 1.143384747 | 0.030469052 |
| ZER1 | 1.13556165 | 0.026554883 |
| C3 | 1.133786846 | 0.030727806 |
| PELI2 | 1.128118316 | 0.049905331 |
| GAB2 | 1.127306966 | 0.014053344 |
| RAB5C | 1.127136337 | 0.046462254 |
| GK | 1.126879128 | 0.009938182 |
| EIF4EBP2 | 1.126835265 | 0.030169208 |
| LINC01159 | 1.124951501 | 0.023733167 |
| ABHD5 | 1.12390728 | 0.017141825 |
| LINC01127 | 1.123302779 | 0.029618731 |
| GSG1L | 1.122962046 | 0.025484592 |
| NLRP12 | 1.122694745 | 0.015586547 |
| TLE3 | 1.122160476 | 0.020347313 |
| HSPC102 | 1.122154361 | 0.01806235 |
| NAV2-AS2 | 1.120879928 | 0.018709182 |
| NT5M | 1.118932112 | 0.0316059 |
| STXBP2 | 1.118843278 | 0.019617116 |
| CCR1 | 1.118173061 | 0.027512045 |
| ANPEP | 1.117128304 | 0.038672876 |
| LIMK2 | 1.116380378 | 0.036158196 |
| HTRA1 | 1.11575009 | 0.033297265 |
| C1QL1 | 1.111031171 | 0.036402664 |
| NDUFV3 | 1.107802365 | 0.020831879 |
| SPINK4 | 1.106191015 | 0.017549644 |
| CBFA2T3 | 1.106075616 | 0.048182703 |
| CD1E | 1.105056569 | 0.038203481 |
| AP2A1 | 1.105049359 | 0.045720132 |
| TMEM95 | 1.104074926 | 0.027946671 |
| TFEB | 1.09903273 | 0.047483021 |
| LOC100506790 | 1.09679236 | 0.04088495 |
| PCP2 | 1.0929485 | 0.039482074 |
| ILDR1 | 1.09050337 | 0.049635387 |
| SLC44A2 | 1.089588387 | 0.046352388 |
| LOC105373334 | 1.089435941 | 0.017564396 |
| FOXE1 | 1.087564841 | 0.041018729 |
| TECPR2 | 1.086043335 | 0.014211867 |
| SGTA | 1.084778107 | 0.043516505 |
| CRACDL | 1.083767386 | 0.017018575 |
| PAGE5 | 1.083209536 | 0.031611886 |
| OBSCN | 1.082025468 | 0.040351114 |
| ARMC5 | 1.07599024 | 0.043075925 |
| LRP10 | 1.07108813 | 0.045296014 |
| TECTA | 1.070417814 | 0.023616757 |
| PLCH2 | 1.069174979 | 0.026152952 |
| RNF26 | 1.066609744 | 0.049337446 |
| ZNF524 | 1.066020255 | 0.033373929 |
| LGALS12 | 1.06355163 | 0.020476704 |
| FZD5 | 1.063442733 | 0.032009981 |
| ZNF467 | 1.062295411 | 0.026339282 |
| SLC66A2 | 1.05761296 | 0.03913557 |
| DPT | 1.057113745 | 0.036970853 |
| SCN5A | 1.056819532 | 0.026310424 |
| FAM171A2 | 1.056227981 | 0.026522308 |
| TIGD5 | 1.050586718 | 0.049577172 |
| TNNI1 | 1.050530455 | 0.048478653 |
| UBA52 | 1.04818494 | 0.018985527 |
| TKT | 1.042514455 | 0.020595489 |
| CYP4F2 | 1.041407391 | 0.030437512 |
| CHRDL2 | 1.039051247 | 0.029995074 |
| TST | 1.035048003 | 0.021558111 |
| CRYBB1 | 1.032554327 | 0.036549498 |
| FAAH | 1.032171273 | 0.03770929 |
| ARAP3 | 1.032055378 | 0.02780745 |
| SDSL | 1.028955403 | 0.02524145 |
| BPI | 1.022495911 | 0.023074181 |
| RARA-AS1 | 1.020647274 | 0.029647411 |
| ARMC6 | 1.019103637 | 0.04136575 |
| SOX4 | 1.014489835 | 0.038184481 |
| CLPB | 1.014264614 | 0.018754751 |
| STK40 | 1.011950551 | 0.027986237 |
| SPAM1 | 1.010335765 | 0.037515007 |
| GADD45A | 1.008349386 | 0.03534894 |
| DSC2 | 1.006663697 | 0.049692635 |
| ZDHHC18 | 1.006266366 | 0.040471182 |
| MEG3 | 1.004989011 | 0.045829531 |
| ARHGAP26 | 1.004449116 | 0.040836976 |
| COX6B1 | 1.004178552 | 0.044405784 |
| ESAM | 1.00373966 | 0.02370027 |
| LOC100130370 | 1.001162217 | 0.045461622 |
| ATXN1L | -1.000568302 | 0.047530517 |
| LINC02273 | -1.000777232 | 0.03340372 |
| ZXDB | -1.006165665 | 0.04500676 |
| OCLN | -1.007808644 | 0.033171057 |
| AGAP4 | -1.011117729 | 0.049989428 |
| RFPL3S | -1.01356987 | 0.038942899 |
| SEC22A | -1.018818125 | 0.032147286 |
| ATF7IP | -1.025603461 | 0.044886229 |
| PPP3CB-AS1 | -1.030973945 | 0.038913108 |
| ZNF320 | -1.035195172 | 0.03610718 |
| ZBTB40 | -1.041438107 | 0.038724078 |
| PPARD | -1.050655697 | 0.030570694 |
| ANKRD55 | -1.055249602 | 0.047702444 |
| LOC100288656 | -1.057826535 | 0.046086656 |
| PTCD2 | -1.058402354 | 0.049010892 |
| LIG1 | -1.059029706 | 0.032510481 |
| ZDHHC21 | -1.061883297 | 0.026787655 |
| LYSMD4 | -1.063982754 | 0.022905221 |
| NOP53 | -1.066139854 | 0.049311968 |
| GNPNAT1 | -1.067621085 | 0.044112479 |
| UNC5CL | -1.068078126 | 0.025226133 |
| FNTB | -1.072817051 | 0.027555953 |
| AGK | -1.076543951 | 0.032881361 |
| ARL5B | -1.078187057 | 0.03557571 |
| NT5E | -1.084746006 | 0.032559954 |
| ZNF614 | -1.090174481 | 0.045740977 |
| RUNDC3B | -1.090858027 | 0.03651919 |
| DPH6 | -1.092275735 | 0.024605929 |
| LOC100287896 | -1.093704735 | 0.025229204 |
| ZNF548 | -1.09576927 | 0.044022509 |
| REC8 | -1.096510323 | 0.039676555 |
| USP53 | -1.0978098 | 0.015628512 |
| DCAF8 | -1.097929271 | 0.046472688 |
| NPTN-IT1 | -1.103378534 | 0.017573876 |
| DTWD1 | -1.107286723 | 0.012023908 |
| ZNF711 | -1.115710298 | 0.011958779 |
| PNMA3 | -1.121307833 | 0.012478593 |
| ATG10 | -1.12352689 | 0.022043529 |
| RAB3IP | -1.129815923 | 0.01415724 |
| PRG4 | -1.129860488 | 0.035857471 |
| CEP126 | -1.131275727 | 0.017779283 |
| ZNF85 | -1.138394572 | 0.029652589 |
| GRAMD2B | -1.139605273 | 0.046859477 |
| DNAJB4 | -1.141161164 | 0.020511685 |
| COPB1 | -1.141746893 | 0.047253156 |
| SLC25A53 | -1.143288996 | 0.023577676 |
| SDHAF4 | -1.144193218 | 0.024166545 |
| NHLRC2 | -1.145775598 | 0.025114778 |
| POLR2J4 | -1.147648926 | 0.027629308 |
| GMNN | -1.150853202 | 0.03160125 |
| MIR4453HG | -1.157661973 | 0.014930697 |
| ZNF667 | -1.160745505 | 0.01589373 |
| ARL6 | -1.162881424 | 0.035740515 |
| YARS2 | -1.164606613 | 0.038625371 |
| BICDL1 | -1.166489382 | 0.034216493 |
| ZNF160 | -1.168196641 | 0.035060009 |
| EXOSC2 | -1.16919725 | 0.040579374 |
| ZGRF1 | -1.173044945 | 0.031384433 |
| SNHG11 | -1.176609205 | 0.046171641 |
| FAM111A-DT | -1.178335681 | 0.021797782 |
| DIPK1A | -1.179238056 | 0.030731574 |
| FAM219B | -1.180822207 | 0.036687266 |
| FRG1BP | -1.18467803 | 0.032449067 |
| TMTC3 | -1.186618227 | 0.010155807 |
| TNKS | -1.188058702 | 0.047432242 |
| E2F6 | -1.190302059 | 0.038901572 |
| NANOG | -1.190528997 | 0.044998261 |
| GPAM | -1.191560021 | 0.020340185 |
| MIR646HG | -1.194179152 | 0.038673012 |
| MTMR9 | -1.194292835 | 0.049777869 |
| GPHN | -1.194566084 | 0.046258075 |
| IRAK1BP1 | -1.198033706 | 0.011771558 |
| OXTR | -1.19839353 | 0.039918224 |
| ITFG2 | -1.200616453 | 0.02123429 |
| RMND1 | -1.203808245 | 0.018628513 |
| RFX3 | -1.20715368 | 0.041802634 |
| HAUS5 | -1.208398845 | 0.028333123 |
| ST8SIA1 | -1.211867107 | 0.029828302 |
| ZNF654 | -1.212683545 | 0.048564965 |
| RIMKLB | -1.217996839 | 0.03626088 |
| PPAT | -1.22220941 | 0.026363967 |
| C1orf109 | -1.222461544 | 0.0162427 |
| NBEA | -1.225029178 | 0.036382433 |
| ZNF587 | -1.225116966 | 0.025195371 |
| ZNF814 | -1.226767574 | 0.008955282 |
| PHF6 | -1.227201126 | 0.045565041 |
| ZNF611 | -1.227960493 | 0.018607281 |
| ZNF134 | -1.231380214 | 0.034590357 |
| ZNF780B | -1.234111875 | 0.014005146 |
| ORC6 | -1.236849353 | 0.020998017 |
| RAB39B | -1.237983431 | 0.04525983 |
| PXYLP1 | -1.24150792 | 0.037817585 |
| G3BP1 | -1.242177192 | 0.044029438 |
| SNX19 | -1.242403006 | 0.026126059 |
| PYHIN1 | -1.243791113 | 0.035038304 |
| GUSBP4 | -1.244841749 | 0.01972726 |
| SLC19A2 | -1.245497908 | 0.036484403 |
| BORA | -1.250897342 | 0.016177729 |
| LOC105378943 | -1.25561411 | 0.040442173 |
| LOC105369477 | -1.256706877 | 0.0151863 |
| TP73-AS1 | -1.260013507 | 0.047892004 |
| ZNF138 | -1.26004912 | 0.045587098 |
| DPY19L2P2 | -1.264058797 | 0.02201214 |
| AHI1 | -1.267936244 | 0.023039685 |
| TENM1 | -1.269921633 | 0.043294273 |
| PHLPP2 | -1.270030193 | 0.040084653 |
| SCN3A | -1.270824732 | 0.044669626 |
| PGAP1 | -1.27565219 | 0.04343407 |
| ZC3H6 | -1.276446901 | 0.026699641 |
| ZNF736 | -1.281219821 | 0.036649586 |
| TRIM5 | -1.281507722 | 0.030527141 |
| FBXO32 | -1.281892256 | 0.036422748 |
| ZFP3 | -1.28408312 | 0.024611591 |
| C8orf37 | -1.285225702 | 0.006982951 |
| MCM3AP-AS1 | -1.287722331 | 0.016677133 |
| GCNT2 | -1.288321411 | 0.022808932 |
| FCGBP | -1.288327772 | 0.04316021 |
| ANXA2R | -1.288564132 | 0.043690113 |
| NADK2 | -1.288782365 | 0.035847101 |
| POC5 | -1.291287698 | 0.042411925 |
| LINC00672 | -1.291969955 | 0.024127452 |
| ERP27 | -1.292913569 | 0.036340524 |
| XPNPEP3 | -1.295300106 | 0.023305572 |
| ARMCX2 | -1.296739985 | 0.02577885 |
| ZNF268 | -1.297380366 | 0.035060846 |
| GPRIN3 | -1.29760891 | 0.027515227 |
| ZNF709 | -1.306480815 | 0.027717879 |
| NBEAL1 | -1.312640302 | 0.038751465 |
| TCEAL1 | -1.315625096 | 0.02343851 |
| MAP7D3 | -1.319189223 | 0.009337574 |
| LOC647070 | -1.320526286 | 0.035906 |
| SETD4 | -1.322286629 | 0.027002121 |
| PYROXD2 | -1.32460032 | 0.034555886 |
| WRN | -1.325612209 | 0.024460642 |
| TCF7 | -1.330898692 | 0.043637712 |
| CFAP44 | -1.332959699 | 0.027709625 |
| HSD17B7 | -1.333017554 | 0.046865264 |
| TC2N | -1.333066322 | 0.043641711 |
| NAIP | -1.334783935 | 0.025454865 |
| LINC02256 | -1.335738671 | 0.03267693 |
| LINC01355 | -1.336616539 | 0.008362484 |
| EP400P1 | -1.34316761 | 0.049291531 |
| LCORL | -1.345312761 | 0.040492201 |
| PTPRK | -1.346894366 | 0.014853594 |
| ZNF300 | -1.348861819 | 0.020226704 |
| LOC100506730 | -1.349050739 | 0.026074123 |
| POGLUT3 | -1.349526114 | 0.024345656 |
| SLC26A11 | -1.351642644 | 0.045059614 |
| SAFB2 | -1.353441353 | 0.02507971 |
| PAXBP1-AS1 | -1.35811921 | 0.0086096 |
| WDPCP | -1.361488692 | 0.032429111 |
| AMMECR1L | -1.363297141 | 0.041220091 |
| C18orf54 | -1.365192688 | 0.009122967 |
| ASXL1 | -1.365428774 | 0.048619921 |
| CCNA2 | -1.368153338 | 0.015430484 |
| NAPB | -1.369592936 | 0.033125458 |
| ZNF571 | -1.371233219 | 0.02156984 |
| RPS6KB1 | -1.371884182 | 0.040671755 |
| ZNF273 | -1.372349632 | 0.04054686 |
| SCAPER | -1.373100704 | 0.025731253 |
| ZBTB26 | -1.37769494 | 0.036851872 |
| TXNDC16 | -1.382060751 | 0.044711574 |
| PGM5-AS1 | -1.382578408 | 0.021594936 |
| FANCI | -1.383289316 | 0.032261514 |
| SENP7 | -1.385734758 | 0.044794431 |
| LOC101928152 | -1.387562575 | 0.034342028 |
| NUTM2B-AS1 | -1.39794343 | 0.015623496 |
| PHOSPHO2 | -1.39822436 | 0.031602299 |
| FZD6 | -1.399291454 | 0.008300023 |
| ALMS1 | -1.399911289 | 0.018573579 |
| UGDH | -1.407911659 | 0.034388827 |
| SNRNP48 | -1.40997224 | 0.005943106 |
| KIF3A | -1.41523366 | 0.025405625 |
| CUZD1 | -1.415245246 | 0.03371058 |
| DZANK1 | -1.420149342 | 0.021071592 |
| NLN | -1.42744458 | 0.028494147 |
| ENOSF1 | -1.433057149 | 0.003845773 |
| FAM184A | -1.433930017 | 0.027249365 |
| NUDCD1 | -1.433979186 | 0.041138804 |
| MAPK8IP3 | -1.4361253 | 0.045940257 |
| TIGD7 | -1.437362225 | 0.024599659 |
| SNHG4 | -1.437940161 | 0.028802862 |
| FKSG49 | -1.441195463 | 0.021934042 |
| TYW5 | -1.443367699 | 0.027802824 |
| MZT2B | -1.448468941 | 0.033703226 |
| ARHGAP5 | -1.4492465 | 0.03500578 |
| ZNF585B | -1.465158091 | 0.04118973 |
| SYNJ2BP | -1.477505516 | 0.036670502 |
| PUS7 | -1.479961608 | 0.040085644 |
| ZNF81 | -1.481078986 | 0.020028421 |
| ABHD18 | -1.488195815 | 0.043203181 |
| DPY19L4 | -1.492429548 | 0.037531667 |
| ZNF681 | -1.499217908 | 0.011121829 |
| GVINP1 | -1.500011001 | 0.014685855 |
| PRKCI | -1.50262639 | 0.049909725 |
| ZNF662 | -1.509360344 | 0.017402339 |
| SMARCAD1 | -1.520393029 | 0.017705355 |
| LOC101929165 | -1.521860995 | 0.025972755 |
| MAP9 | -1.525890073 | 0.030832777 |
| ZNF284 | -1.527851002 | 0.040337146 |
| PEX3 | -1.530773838 | 0.037952988 |
| GOLGA8N | -1.533174023 | 0.041685477 |
| LOC105376486 | -1.538035339 | 0.010328863 |
| ACER3 | -1.538621928 | 0.038066797 |
| ZNF823 | -1.545523149 | 0.00666154 |
| LINC01550 | -1.54938865 | 0.008427262 |
| BIRC3 | -1.550198067 | 0.045177049 |
| NAPEPLD | -1.568866945 | 0.029886968 |
| HACE1 | -1.578822503 | 0.016165954 |
| PIAS2 | -1.57931628 | 0.018019786 |
| ODR4 | -1.584958942 | 0.02718596 |
| AGBL3 | -1.586925903 | 0.004779461 |
| SCML1 | -1.589204734 | 0.044806362 |
| RSKR | -1.592176384 | 0.045414879 |
| ZNF677 | -1.59670419 | 0.014504077 |
| ZKSCAN7 | -1.604459689 | 0.016083801 |
| TBC1D32 | -1.608746127 | 0.013203158 |
| EME2 | -1.61069338 | 0.027303946 |
| MASTL | -1.611018938 | 0.008988766 |
| NRCAM | -1.617792791 | 0.008713442 |
| STMN3 | -1.623229907 | 0.011360592 |
| KLHDC1 | -1.63027131 | 0.014961648 |
| MRI1 | -1.633830316 | 0.028243403 |
| HOOK1 | -1.640403811 | 0.009334694 |
| TBCEL | -1.646242838 | 0.035707564 |
| MICU3 | -1.647949906 | 0.005304431 |
| ERVK13-1 | -1.65265006 | 0.020524634 |
| ZC3H8 | -1.659113002 | 0.028204933 |
| ALDH8A1 | -1.667864698 | 0.009355628 |
| LOC285097 | -1.670576784 | 0.006108375 |
| AGMAT | -1.68175993 | 0.018250787 |
| LRCH3 | -1.68727033 | 0.044468717 |
| SLC25A16 | -1.694153852 | 0.018886098 |
| SLC33A1 | -1.69425201 | 0.040477017 |
| LOC100289333 | -1.697329203 | 0.011577909 |
| HLTF | -1.706088552 | 0.042498924 |
| C5orf24 | -1.70615139 | 0.021676696 |
| LOC389765 | -1.708633106 | 0.016094028 |
| PWAR6 | -1.710254729 | 0.026429947 |
| SEPSECS | -1.726976287 | 0.034567766 |
| SRGAP2B | -1.728232154 | 0.039099638 |
| GPATCH2 | -1.737468067 | 0.003560274 |
| EPM2AIP1 | -1.738322559 | 0.023140303 |
| TMEM260 | -1.746737928 | 0.0282504 |
| PLAG1 | -1.747763845 | 0.010117837 |
| MPHOSPH10 | -1.752812936 | 0.049220301 |
| ABCD3 | -1.762050018 | 0.024837068 |
| MAP4K3 | -1.762112609 | 0.03015512 |
| LOC389834 | -1.764722262 | 0.022845287 |
| PAX5 | -1.765886136 | 0.01673461 |
| ZNF708 | -1.769760134 | 0.04816823 |
| MIR3682 | -1.770964454 | 0.019196754 |
| GORAB | -1.774747682 | 0.048376727 |
| MYEF2 | -1.777835112 | 0.007480107 |
| LINC01410 | -1.78120013 | 0.017934164 |
| ZNF550 | -1.783481893 | 0.011538886 |
| ARL10 | -1.795155003 | 0.010436372 |
| EXTL2 | -1.795822836 | 0.013599238 |
| YES1 | -1.800137219 | 0.020746381 |
| CEP43 | -1.801428972 | 0.032653237 |
| SNHG21 | -1.80501366 | 0.015411105 |
| SCAI | -1.806176299 | 0.036995154 |
| HAUS6 | -1.829123725 | 0.047478838 |
| LOC202181 | -1.837657421 | 0.003130177 |
| SRSF11 | -1.889884451 | 0.021502548 |
| BTAF1 | -1.892086423 | 0.034456909 |
| SPIN3 | -1.894886579 | 0.003169422 |
| KANSL1L | -1.897952861 | 0.000995046 |
| ANKRD36BP2 | -1.94274593 | 0.01939476 |
| LEF1-AS1 | -1.984092614 | 0.003380259 |
| ZNF529 | -2.027167862 | 0.023485358 |
| RASEF | -2.049061735 | 0.014065544 |
| GPRASP1 | -2.06322535 | 0.03452894 |
| ZNF566 | -2.06521893 | 0.010004356 |
| NAP1L3 | -2.073681428 | 0.009268111 |
| MSANTD2 | -2.077234844 | 0.043875474 |
| LOC105369228 | -2.079352118 | 0.013295645 |
| ZC3H12D | -2.084981718 | 0.047586589 |
| CEP290 | -2.088944613 | 0.037245991 |
| HELLS | -2.113198176 | 0.009292809 |
| LINC02754 | -2.152296837 | 0.001908325 |
| SLC6A16 | -2.159743536 | 0.002100734 |
| ZNF738 | -2.179262568 | 0.016377574 |
| TUBE1 | -2.194129751 | 0.04520477 |
| FAM169A | -2.213702679 | 0.005109356 |
| RBM4 | -2.240922177 | 0.021342605 |
| AHSA2P | -2.37363599 | 0.036191435 |
| LOC100190986 | -2.421165358 | 0.041271467 |
| NFATC2IP | -2.542708176 | 0.01234198 |
| CENATAC | -2.570407667 | 0.011585438 |
| PRKXP1 | -2.626501731 | 0.015264556 |
| FRG1JP | -2.647474087 | 0.00012907 |
| ZMAT1 | -2.664477131 | 0.020037544 |
| CENPK | -3.13636212 | 0.016681303 |
| Abbreviations: DEmRNAs, differentially expressed mRNAs; CAD, coronary artery disease; FC, fold change. | | |

| **Table S4:** GO Enrichment analysis of DEmRNAs. | | | |
| --- | --- | --- | --- |
| Identification | Term | Count | *P-*Value |
| **GO-BP** |  |  |  |
| GO:0006955 | immune response | 35 | 1.60E-05 |
| GO:0006954 | inflammatory response | 32 | 2.95E-05 |
| GO:0032496 | response to lipopolysaccharide | 18 | 1.18E-04 |
| GO:0015671 | oxygen transport | 6 | 1.47E-04 |
| GO:0043123 | positive regulation of I-kappaB kinase/NF-kappaB signaling | 17 | 3.00E-04 |
| GO:0008285 | negative regulation of cell proliferation | 30 | 3.56E-04 |
| GO:0006098 | pentose-phosphate shunt | 5 | 4.88E-04 |
| GO:0006935 | chemotaxis | 14 | 5.57E-04 |
| GO:0050900 | leukocyte migration | 14 | 5.57E-04 |
| GO:0050790 | regulation of catalytic activity | 9 | 0.002817717 |
| GO:0030593 | neutrophil chemotaxis | 9 | 0.002817717 |
| GO:0030218 | erythrocyte differentiation | 7 | 0.003204122 |
| GO:0046597 | negative regulation of viral entry into host cell | 5 | 0.003682314 |
| GO:0055072 | iron ion homeostasis | 6 | 0.004411636 |
| GO:0042744 | hydrogen peroxide catabolic process | 5 | 0.005500214 |
| GO:0006351 | transcription, DNA-templated | 93 | 0.007538387 |
| GO:0038094 | Fc-gamma receptor signaling pathway | 3 | 0.007722725 |
| GO:0000209 | protein polyubiquitination | 15 | 0.008223888 |
| GO:0001836 | release of cytochrome c from mitochondria | 5 | 0.009213724 |
| GO:0045087 | innate immune response | 27 | 0.009326298 |
| GO:0007204 | positive regulation of cytosolic calcium ion concentration | 12 | 0.010634324 |
| GO:0034341 | response to interferon-gamma | 5 | 0.010740856 |
| GO:0001817 | regulation of cytokine production | 4 | 0.010765572 |
| GO:0034612 | response to tumor necrosis factor | 5 | 0.01242224 |
| GO:0008654 | phospholipid biosynthetic process | 6 | 0.015084862 |
| GO:0048821 | erythrocyte development | 4 | 0.016217102 |
| GO:0043122 | regulation of I-kappaB kinase/NF-kappaB signaling | 4 | 0.016217102 |
| GO:0006355 | regulation of transcription, DNA-templated | 72 | 0.017199006 |
| GO:0043066 | negative regulation of apoptotic process | 27 | 0.017939005 |
| GO:0020027 | hemoglobin metabolic process | 3 | 0.01838106 |
| GO:0032364 | oxygen homeostasis | 3 | 0.01838106 |
| GO:0009052 | pentose-phosphate shunt, non-oxidative branch | 3 | 0.01838106 |
| GO:0061418 | regulation of transcription from RNA polymerase II promoter in response to hypoxia | 5 | 0.018440089 |
| GO:0035666 | TRIF-dependent toll-like receptor signaling pathway | 5 | 0.018440089 |
| GO:0007568 | aging | 13 | 0.019078401 |
| GO:0031623 | receptor internalization | 6 | 0.020178817 |
| GO:0015701 | bicarbonate transport | 6 | 0.022098354 |
| GO:0007249 | I-kappaB kinase/NF-kappaB signaling | 7 | 0.022597688 |
| GO:0045454 | cell redox homeostasis | 8 | 0.02305751 |
| GO:0030219 | megakaryocyte differentiation | 3 | 0.025111908 |
| GO:0006769 | nicotinamide metabolic process | 3 | 0.025111908 |
| GO:0006914 | autophagy | 11 | 0.025145624 |
| GO:0045893 | positive regulation of transcription, DNA-templated | 29 | 0.025891801 |
| GO:0006977 | DNA damage response, signal transduction by p53 class mediator resulting in cell cycle arrest | 7 | 0.026109127 |
| GO:0050727 | regulation of inflammatory response | 7 | 0.027993383 |
| GO:0070936 | protein K48-linked ubiquitination | 6 | 0.028553065 |
| GO:0043065 | positive regulation of apoptotic process | 19 | 0.029349033 |
| GO:0002755 | MyD88-dependent toll-like receptor signaling pathway | 5 | 0.031932051 |
| GO:0042119 | neutrophil activation | 3 | 0.032676375 |
| GO:0014070 | response to organic cyclic compound | 6 | 0.033454616 |
| GO:0046697 | decidualization | 4 | 0.035482865 |
| GO:0007266 | Rho protein signal transduction | 6 | 0.036089426 |
| GO:0043161 | proteasome-mediated ubiquitin-dependent protein catabolic process | 14 | 0.037654012 |
| GO:0006641 | triglyceride metabolic process | 5 | 0.038594341 |
| GO:0000422 | mitophagy | 5 | 0.038594341 |
| GO:0042542 | response to hydrogen peroxide | 6 | 0.038848489 |
| GO:0048013 | ephrin receptor signaling pathway | 8 | 0.039006877 |
| GO:0007596 | blood coagulation | 13 | 0.03973217 |
| GO:0006783 | heme biosynthetic process | 4 | 0.040296738 |
| GO:1901223 | negative regulation of NIK/NF-kappaB signaling | 3 | 0.041004192 |
| GO:0006596 | polyamine biosynthetic process | 3 | 0.041004192 |
| GO:0034097 | response to cytokine | 6 | 0.04173274 |
| GO:0030177 | positive regulation of Wnt signaling pathway | 5 | 0.04220086 |
| GO:0098869 | cellular oxidant detoxification | 7 | 0.043700384 |
| GO:2001243 | negative regulation of intrinsic apoptotic signaling pathway | 4 | 0.045422226 |
| GO:0090023 | positive regulation of neutrophil chemotaxis | 4 | 0.045422226 |
| **GO-CC** |  |  |  |
| GO:0005833 | hemoglobin complex | 7 | 1.75E-06 |
| GO:0005829 | cytosol | 159 | 1.32E-04 |
| GO:0005622 | intracellular | 74 | 2.92E-04 |
| GO:0030863 | cortical cytoskeleton | 6 | 9.94E-04 |
| GO:0070062 | extracellular exosome | 133 | 0.001007096 |
| GO:0031225 | anchored component of membrane | 11 | 0.008263568 |
| GO:0005774 | vacuolar membrane | 4 | 0.012979058 |
| GO:0031307 | integral component of mitochondrial outer membrane | 4 | 0.039300209 |
| GO:0005741 | mitochondrial outer membrane | 11 | 0.046211441 |
| **GO-MF** |  |  |  |
| GO:0005344 | oxygen transporter activity | 6 | 1.03E-04 |
| GO:0005515 | protein binding | 371 | 1.23E-04 |
| GO:0031625 | ubiquitin protein ligase binding | 24 | 4.38E-04 |
| GO:0019864 | IgG binding | 5 | 4.96E-04 |
| GO:0019825 | oxygen binding | 8 | 0.00158429 |
| GO:0003676 | nucleic acid binding | 54 | 0.003874063 |
| GO:0020037 | heme binding | 13 | 0.004871896 |
| GO:0016874 | ligase activity | 20 | 0.005638718 |
| GO:0046872 | metal ion binding | 99 | 0.005643035 |
| GO:0042277 | peptide binding | 8 | 0.005875102 |
| GO:0003700 | transcription factor activity, sequence-specific DNA binding | 51 | 0.0091355 |
| GO:0004842 | ubiquitin-protein transferase activity | 22 | 0.010950643 |
| GO:0003714 | transcription corepressor activity | 15 | 0.018856304 |
| GO:0048029 | monosaccharide binding | 3 | 0.025319187 |
| GO:0030506 | ankyrin binding | 4 | 0.035879424 |
| GO:0003677 | DNA binding | 76 | 0.045276252 |
| Abbreviations: GO, gene ontology; DEmRNAs, differentially expressed mRNAs; BP, biological process; CC, cellular component; MF, molecular function. | | | |

| **Table S5:** KEGG pathway Enrichment analysis of DEmRNAs. | | | |
| --- | --- | --- | --- |
| Identification | Term | Count | *P-*Value |
| **KEGG** |  |  |  |
| hsa04064 | NF-kappa B signaling pathway | 11 | 0.002035606 |
| hsa00760 | Nicotinate and nicotinamide metabolism | 6 | 0.004914041 |
| hsa04380 | Osteoclast differentiation | 12 | 0.01361295 |
| hsa04060 | Cytokine-cytokine receptor interaction | 18 | 0.014613776 |
| hsa05120 | Epithelial cell signaling in Helicobacter pylori infection | 8 | 0.015459564 |
| hsa05150 | Staphylococcus aureus infection | 7 | 0.018404925 |
| hsa01100 | Metabolic pathways | 62 | 0.022117992 |
| hsa04668 | TNF signaling pathway | 10 | 0.024232305 |
| hsa00030 | Pentose phosphate pathway | 5 | 0.025450864 |
| hsa05202 | Transcriptional misregulation in cancer | 13 | 0.030957451 |
| hsa05014 | Amyotrophic lateral sclerosis (ALS) | 6 | 0.045167345 |
| hsa05152 | Tuberculosis | 13 | 0.045253108 |
| Abbreviations: KEGG, Kyoto Encyclopedia of Genes and Genomes. KEGG; DEmRNAs, differentially expressed mRNAs. | | | |

| **Table S6:** Complete list of circRNA-miRNA-hubgene subnetwork pairs. | | |
| --- | --- | --- |
| circRNA | miRNA | hubgene |
| hsa_circ_0085340 | hsa-miR-136-5p | UBE2D1 |
| hsa_circ_0085340 | hsa-miR-411-5p | UBE2D1 |
| hsa_circ_0085340 | hsa-miR-136-5p | UBE2F |
| hsa_circ_0085340 | hsa-miR-136-5p | UBE2H |
| hsa_circ_0085340 | hsa-miR-136-5p | MKRN1 |
| hsa_circ_0085340 | hsa-miR-136-5p | UBE2W |
| hsa_circ_0085340 | hsa-miR-411-5p | UBE2W |
| hsa_circ_0071330 | hsa-miR-411-5p | UBE2D1 |
| hsa_circ_0071330 | hsa-miR-411-5p | UBE2W |
| hsa_circ_0071326 | hsa-miR-376c-3p | SIAH2 |
| hsa_circ_0071326 | hsa-miR-376c-3p | UBE2J1 |
| hsa_circ_0071326 | hsa-miR-376c-3p | MKRN1 |
| hsa_circ_0069094 | hsa-miR-136-5p | UBE2D1 |
| hsa_circ_0069094 | hsa-miR-136-5p | UBE2F |
| hsa_circ_0069094 | hsa-miR-136-5p | UBE2H |
| hsa_circ_0069094 | hsa-miR-136-5p | MKRN1 |
| hsa_circ_0069094 | hsa-miR-136-5p | UBE2W |
| hsa_circ_0047336 | hsa-miR-376c-3p | SIAH2 |
| hsa_circ_0047336 | hsa-miR-376c-3p | UBE2J1 |
| hsa_circ_0047336 | hsa-miR-376c-3p | MKRN1 |
| hsa_circ_0045512 | hsa-miR-136-5p | UBE2D1 |
| hsa_circ_0045512 | hsa-miR-136-5p | UBE2F |
| hsa_circ_0045512 | hsa-miR-136-5p | MKRN1 |
| hsa_circ_0045512 | hsa-miR-136-5p | UBE2H |
| hsa_circ_0045512 | hsa-miR-136-5p | UBE2W |
| hsa_circ_0020417 | hsa-miR-136-5p | UBE2D1 |
| hsa_circ_0020417 | hsa-miR-136-5p | MKRN1 |
| hsa_circ_0020417 | hsa-miR-136-5p | UBE2F |
| hsa_circ_0020417 | hsa-miR-136-5p | UBE2H |
| hsa_circ_0020417 | hsa-miR-136-5p | UBE2W |
| hsa_circ_0020417 | hsa-miR-654-5p | UBE2J1 |
| hsa_circ_0020417 | hsa-miR-654-5p | ZNRF1 |
| hsa_circ_0020387 | hsa-miR-136-5p | UBE2D1 |
| hsa_circ_0020387 | hsa-miR-136-5p | MKRN1 |
| hsa_circ_0020387 | hsa-miR-136-5p | UBE2F |
| hsa_circ_0020387 | hsa-miR-136-5p | UBE2H |
| hsa_circ_0020387 | hsa-miR-136-5p | UBE2W |
| hsa_circ_0020275 | hsa-miR-136-5p | UBE2D1 |
| hsa_circ_0020275 | hsa-miR-136-5p | UBE2F |
| hsa_circ_0020275 | hsa-miR-136-5p | MKRN1 |
| hsa_circ_0020275 | hsa-miR-136-5p | UBE2H |
| hsa_circ_0020275 | hsa-miR-136-5p | UBE2W |
| Abbreviations: circRNA, circular RNA; miRNA, microRNA. | | |

| **Table S7:** Functional enrichment analysis of hubgenes. | | | |
| --- | --- | --- | --- |
| Identification | Term | Count | *P-*Value |
| **GO-BP** |  |  |  |
| GO:0043161 | proteasome-mediated ubiquitin-dependent protein catabolic process | 5 | 3.05E-07 |
| GO:0000209 | protein polyubiquitination | 4 | 2.53E-05 |
| GO:0070936 | protein K48-linked ubiquitination | 3 | 1.14E-04 |
| GO:0006511 | ubiquitin-dependent protein catabolic process | 3 | 0.001703013 |
| GO:0070979 | protein K11-linked ubiquitination | 2 | 0.009610179 |
| **GO-CC** |  |  |  |
| GO:0005737 | cytoplasm | 5 | 0.02596441 |
| **GO-MF** |  |  |  |
| GO:0004842 | ubiquitin-protein transferase activity | 6 | 1.61E-08 |
| GO:0061630 | ubiquitin protein ligase activity | 5 | 2.15E-07 |
| GO:0016874 | ligase activity | 4 | 7.81E-05 |
| GO:0031625 | ubiquitin protein ligase binding | 3 | 0.004129942 |
| GO:0061631 | ubiquitin conjugating enzyme activity | 2 | 0.010264793 |
| GO:0005524 | ATP binding | 4 | 0.011298559 |
| GO:0005515 | protein binding | 7 | 0.019847501 |
| **KEGG** |  |  |  |
| hsa05322 | Ubiquitin mediated proteolysis | 4 | 7.73E-06 |
| Abbreviations: GO, gene ontology; BP, biological process; CC, cellular component; MF, molecular function; KEGG, Kyoto Encyclopedia of Genes and Genomes.KEGG, Kyoto Encyclopedia of Genes and Genome. | | | |
